# Attenuating α-synuclein pathology in mice with in situ engineered astrocytes

**DOI:** 10.1186/s40035-025-00518-0

**Published:** 2025-11-20

**Authors:** Xiao-yu Du, Jing Zhou, Jie Zhu, Lun Zhang, Shuai Lu, Shi-yu Liang, Fang Cui, Hao-han Zhang, Fei Chen, Ming-yue Jiao, Ya-ru Huang, Xiao-lin Yu, Rui-tian Liu

**Affiliations:** 1 National Key Laboratory of Biochemical Engineering, Institute of Process Engineering, Chinese Academy of Sciences, Haidian District, Beijing, 100190 China; 2 University of Chinese Academy of Sciences, Beijing, 100049 China; 3 Department of Geriatric Rehabilitation, Beijing Rehabilitation Hospital, Capital Medical University, Beijing, 100144 China; 4 Ningxia University, Yinchuan, 750021 China

**Keywords:** Parkinson’s disease, α-Synuclein oligomer, Astrocyte, Chimeric antigen receptor

## Abstract

**Background:**

α-Synuclein oligomers (α-synOs) contribute to the initiation and progression of Parkinson’s disease (PD) by promoting neuronal death and activating glial cells. Clearing α-synOs while maintaining tissue homeostasis is a promising therapeutic strategy for PD.

**Methods:**

We genetically engineered astrocytes with an anti-α-synO chimeric antigen receptor (CAR) consisting of a single-chain variable fragment targeting α-synOs and a truncated MerTK receptor, to direct their phagocytic activity against α-synOs.

**Results:**

CAR-engineered astrocytes (CAR-A) showed significantly enhanced phagocytosis of α-synOs due to effective activation of Rac1, Cdc42 and RhoA and markedly decreased the release of pro-inflammatory cytokines by inhibiting the NF-κB and cytokine receptor signaling pathways. Consistently, in situ CAR-A significantly ameliorated the motor and cognitive deficits of A53T mice by clearing α-synOs, creating a non-inflammatory microenvironment and restoring the viability of dopaminergic neurons.

**Conclusions:**

CAR-A-based strategy is an effective treatment for PD-like mouse model. This in situ CAR-A technology provides an innovative and feasible strategy to treat PD and other brain disorders.

**Supplementary Information:**

The online version contains supplementary material available at 10.1186/s40035-025-00518-0.

## Background

α-Synuclein oligomers (α-synOs) play a critical role in the pathogenesis, motor dysfunction and non-motor deficits of Parkinson’s disease (PD) by inducing substantia nigra dopaminergic neurodegeneration and striatal dopamine loss [1]. Extracellular α-synOs secreted by neurons can be taken up by other neurons and glial cells, resulting in the replication and propagation of pathological α-syn aggregates in neurons and glial inflammatory responses under pathological conditions [1–3]. Therefore, clearing α-synOs while maintaining tissue homeostasis is a promising therapeutic strategy for PD. Astrocytes are the most abundant glial cells in the central nervous system (CNS), which have a wide range of essential functions for CNS [4]. However, pathologic α-synOs convert astrocytes into a reactive phenotype. The reactive astrocytes release a variety of neurotoxic chemokines and cytokines, and lose their normal function in maintaining synapse formation and function, as well as phagocytosing abnormal aggregated proteins such as α-synOs [5–7], mainly contributing to PD progression. Therefore, reversing disabled and deleterious astrocytes to the normal phenotype holds therapeutic potential for PD.

Previous studies have used bispecific antibody or GAS6 fusion protein to achieve anti-inflammatory clearance of protein aggregates via TAM (Tyro3, Axl, and Mer tyrosine kinase [MerTK]) receptors [8, 9]. In this study, instead of using antibody-mediated therapy, we designed a chimeric antigen receptor expressed on astrocytes (CAR-A) to clear extracellular α-synOs. As activation of phagocytic acceptors such as Toll-like receptors on astrocytes induces the release of inflammatory factors [10, 11], our chimeric antigen receptor (CAR) was constructed based on MerTK, a phagocytic receptor mainly expressed on anti-inflammatory macrophages and astrocytes, to achieve phagocytosis and simultaneous inhibition of cytokine release upon activation [12–14]. Moreover, we replaced the ligand-binding domain of MerTK with an α-synO-specific single-chain variable fragment 3A, to achieve the targeting of α-synOs. 3A was isolated from a single-chain variable fragment (scFv) library by phage display, and has been improved through affinity maturation to specifically bind α-synOs rather than α-syn monomers and fibrils. The function and the therapeutic effect of the CAR-A were then detected in vitro, and in A53T PD transgenic mice.

## Materials and Methods

### Primary microglial and astrocyte cultures

Primary astrocyte and microglia cultures were prepared from C57BL/6 mice as described previously [15]. Briefly, brain cortices from 10 postnatal mice were dissected in Dulbecco’s PBS (dPBS) and the meninges were removed. The tissues were enzymatically dissociated with papain enzyme at 5% CO_2_ and then mechanically dissociated to produce single cell suspension. For astrocyte isolation, after removing microglia and oligodendrocyte precursor cell (OPCs) through immunopanning dishes coated with anti-BSL1, anti-CD11b and anti-O4 antibodies, 100 μL of 0.5 mg/mL mouse anti-ITGB5 (ThermoFisher Scientific, Waltham, MA, #14-0497-82, RRID: AB_467288) was added into 5–10 mL of cell suspension and incubated for 30–40 min at 37 °C. The cell suspensions were allowed to interact with the immunopanning dish (coated with 60 μL of anti-mouse IgG in 50 mmol/L Tris–HCl pH 9.5 overnight at 4℃) for 20 min at room temperature. The unbound cells and debris were removed by washing the dish with dPBS for 10 times, and freshly isolated astrocytes were cultured in DMEM/Neurobasal (1:1) medium containing 100 units/mL penicillin, 100 μg/mL streptomycin (ThermoFisher Scientific,15140122), 1 mmol/L sodium pyruvate (ThermoFisher Scientific, 11360070), 292 μg/mL L-glutamine (ThermoFisher Scientific, 25030081), 1 μg/mL transferrin (Sigma, St. Louis, MO, T8158), 0.16 μg/mL putrescine (Sigma, P5780), 1 nmol/L progesterone (Merck, Darmstadt, Germany, P0130), 0.4 ng/mL sodium selenite (ThermoFisher Scientific), 5 μg/mL N-Acetyl-L-cysteine (Sigma, A8199) and 5 ng/mL HBEGF (MedChemExpress, Shanghai, China, #HY-P7400). For microglia isolation, cell suspensions were applied directly to positive-selection immunopanning dishes coated with anti-CD11b monoclonal antibodies. The freshly isolated microglia were cultured in DMEM/F12 medium containing 100 units/mL penicillin, 100 μg/mL streptomycin, 2 mmol/L L-glutamine, 5 μg/mL *N*-acetyl cysteine, 5 μg/mL insulin (Merck, I9278), 100 μg/mL transferrin, and 100 ng/mL sodium selenite.

### Primary neuronal cultures

Primary neurons were obtained from 14 to 15-day-old embryos of C57BL/6 mice. Briefly, hippocampi were dissected from the brains and incubated with trypsin and DNase I before careful trituration to generate single-cell suspensions. The cells were plated on poly-D-lysine-coated coverslips at a density of 300,000 cells/well in a 12-well dish and cultured in neurobasal medium (Gibco, Waltham, MA, #21103049) with B27 (Gibco, #17504–044) and l-GlutaMAX (Gibco, #35050061). The medium was replaced every 2–3 days, and primary neurons were cultured for 2 weeks for subsequent experiments.

### Plasmid construction

All of the plasmids used in this project were custom-cloned by Sangon Biotech Co., Ltd (Shanghai, China). The following 3 gene expression vectors were used:

(1) pGfaABC1D-3A-^276-994^MerTK-EGFP-BGH polyA, (2) pGfaABC1D-ns-scFv-^276-994^MerTK-EGFP-BGH polyA, and (3) pGfaABC1D-3A-^276-994^MerTK-BGH polyA.

In the first construct, the enhanced CMV promotor sequence in the MerTK expression plasmid (purchased from Sino Biological Inc., #MG50514-ACG) was replaced with GfaABC1D promotor sequence synthesized from Sangon. A signal peptide sequence was inserted before an α-synO scFv 3A to achieve membrane targeting. The ligand-binding domain gene corresponding to amino acids 19–275 of MerTK was replaced with 3A gene sequence. To facilitate the detection of receptor, we co-expressed enhanced green fluorescent protein (EGFP) along with the ^276-994^MerTK before the stop codon and the bovine growth hormone (BGH) poly-A signal. Here, 3A was isolated from an scFv library by phage display, and improved through affinity maturation to specifically bind α-synOs, but not α-syn monomers and fibrils.

In the second construct, the 3A single-chain variable fragment gene sequence in plasmid 1 was replaced with a nonspecific single-chain variable fragment (ns-scFv) gene sequence, which has been reported by Aburatani et al. [16].

In the third construct, EGFP gene sequence in plasmid 1 was deleted.

### Thioflavin T (ThT) fluorescence assay

The assay was performed by periodically adding 198 μL of 5 mmol/L ThT in 50 mmol/L phosphate buffer (pH 6.5) to 2 μL samples, and the ThT fluorescence intensity was measured using a Tecan Safire2 microplate reader (Tecan, Switzerland) set to 450 nm/482 nm (excitation/emission).

### α-Syn purification and α-syn preformed fibril (PFF) preparation

α-Syn was expressed and prepared using the method described previously [17]. α-SynOs were prepared as previously described with some modifications [18]. Briefly, freshly prepared α-syn was diluted to 1 mg/mL with PBS, then incubated at 37 °C under constant stirring at 200 rpm. The aggregation state of α-syn was determined by ThT fluorescence assay and native-PAGE assay. The incubation time for the preparation of α-synOs was 72 h. For fibril preparation, α-syn monomers were solubilized in PBS at the concentration of 5 mg/mL and incubated at 37 °C with shaking for 7 days [19]. Then, the PFF-containing solution was centrifuged at 13,000× *g* for 10 min, the supernatant discarded, and the precipitate resuspended. These steps were repeated twice to remove soluble monomers and oligomers as well as unstable fibers from the solution. Transmission electron microscopy (TEM) was used to characterize PFF at an accelerating voltage of 100 kV (HITACHI, HT7700, Japan). Then, the fibrils were sonicated 10 min using a water bath sonicator. To verify the seed capacity of α-syn PFF, PFF sonicated for different times were added to 1 mg/mL α-syn monomers at a mass ratio of 1:20 and incubated at 37 °C under constant stirring as described previously [20]. The aggregation state of α-syn was determined by ThT fluorescence assay. After 24 h of incubation, the solution was centrifuged at 17,500 × *g* for 20 min, and the concentration of the supernatant was determined.

### Flow cytometry

CAR-A with CAR expression and α-synO phagocytosis were tested by flow cytometry using a two-step staining protocol as previously described with some modifications [21]. Briefly, after fixation in 4% paraformaldehyde (PFA) for 20 min, transfected astrocytes were treated with 0.3% Triton X-100, and stained with anti-α-syn primary antibody (Abcam, #ab138501, RRID: AB_2537217), followed by donkey anti-rabbit antibody (Biolegend, #406421, RRID: AB_2563484) staining. Flow cytometry data were acquired on a CytoFLEX LX (Beckman, Brea, CA) and analyzed with FlowJo X10 (FlowJo, RRID: SCR_008520).

### Immunocytochemistry analysis

Cells cultured on glass slides were fixed with 4% PFA for 20 min at room temperature, blocked in blocking buffer (10% donkey serum in PBS + 0.3% Triton- X100) for 1 h, incubated with primary antibodies overnight at 4 °C followed by fluorescently conjugated secondary antibodies for 1 h at room temperature in the dark, and mounted on coverslips with antifade mounting medium (Solarbio, Beijing, China, #S2110). To detect cell apoptosis, fluorescent terminal deoxynucleotidyl transferase nick-end labeling (TUNEL) kit was used according to the manufacturer’s protocols (Beyotime, Shanghai, China, #C1090). Fluorescence signals were captured on a laser scanning confocal microscope (Leica, Mannheim, Germany, TCS SP8). Primary antibodies for the following proteins were used: MerTK (Abcam, #ab95925, RRID: AB_10863559, 1:100), 3A (monoclonal antibody, prepared by our laboratory, 1:100), α-syn (Abcam, #ab138501, RRID: AB_2537217, 1:100; BD biosciences, Franklin Lakes, NJ, #610787, RRID: AB_398108, 1:100), pS129 α-syn (Cell Signaling Technology, Danvers, MA, #23706, RRID: AB_2798868, 1:100), Lamp1 (Abcam, #ab24170, RRID: AB_775978, 1:100), ionized calcium binding adaptor molecule 1 (Iba-1) (GeneTex, Irvine, TX, #GTX100042, RRID: AB_1240434, 1:100); microtubule-associated protein 2 (MAP2) (Abcam, #ab32454, RRID: AB_776174, 1:100), glial fibrillary acidic protein (GFAP) (Cell Signaling Technology, #3670S, RRID: AB_561049, 1:100; Affinity, Nanjing, China, #DF6040, AB_2838012, 1:100; Abcam, #ab302644, 1:100), C3 (Abcam, #ab97462, RRID: AB_10679468, 1:100), and S100β (SinoBiological, Beijing, China, #100508-MM30, 1:100). Secondary antibodies used in this study included: donkey anti-mouse IgG H&L (Alexa Fluor® 488; Abcam, Cat. #ab150105; 1:500), donkey anti-mouse IgG H&L (Alexa Fluor® 555; Abcam, Cat. #ab150110; 1:500), donkey anti-rabbit IgG H&L (Alexa Fluor® 488; Abcam, Cat. #ab150073; 1:500), donkey anti-rabbit IgG H&L (Alexa Fluor® 647; Abcam, Cat. #ab150063; 1:500).

### Western blotting and dot blotting analysis

For western blotting analysis, protein samples from brain lysates and cell lysates were electrophoresed on 8% or 12% SDS-PAGE and transferred to nitrocellulose membranes. When Native-PAGE was used for electrophoresis, protein samples were electrophoresed on 3%–8% Tris–Acetate PAGE (Beyotime, #P0539S) using sample loading buffer and electrophoresis buffer without SDS. For the characterization of α-syn monomers, oligomers, and PFFs, different aggregation forms of α-syn were diluted using lysis buffer (10 mmol/L Tris–HCl, pH 7.4, 150 mmol/L NaCl, 5 mmol/L EDTA, 1% Triton X-100). The solution was centrifuged at 17,500 × *g* for 20 min at 4 °C. The supernatant obtained after centrifugation represented the Triton X-100-soluble fraction. The pellet was washed once with lysis buffer, and the resulting pellet was homogenized in lysis buffer containing 1% SDS. The homogenate was centrifuged, and the resulting supernatant was the Triton X-100-insoluble fraction.

For dot blotting analysis, protein samples were dropped on nitrocellulose membranes. After blocking with 5% nonfat milk for 1 h at room temperature, the membrane was probed overnight at 4 °C with a primary antibody. After washing with PBS containing 0.1% Tween-20 (PBST), HRP-conjugated secondary antibodies were used at a concentration of 1:5000 at room temperature. The bands in immunoblots were visualized by enhanced chemiluminescence using an Amersham imager 680 imaging system (GE Healthcare, Chicago, IL) and quantified by the Image J software (RRID: SCR_003070). Separation of membrane and cytosol proteins was performed using the Membrane and Cytosol Protein Extraction Kit (Beyotime, #P0033) according to the manufacture’s instructions. Primary antibodies for the following proteins were used: Ras homolog family member A (RhoA) (Invitrogen, #PA5-87403, RRID: AB_2804123, 1:500), Rac1 (Invitrogen, #PA1-091X, RRID: AB_2539857, 1:500), cell division cycle 42 (Cdc42) (Invitrogen, #PA1-092, RRID: AB_2539858, 1:500), suppressor of cytokine signaling (SOCS) 1 (Sangon, #D260748, 1:1000), SOCS3 (Sangon, #D121242, 1:1000), p65 (Cell Signaling Technology, #8242, RRID: AB_10859369, 1:1000), MerTK (Abcam, #ab95925, RRID: AB_10863559, 1:1000), pMerTK (Bioss, Beijing, China, #bs18791R, 1:1000), pStat-1 (Beyotime, #AF5935), IκB-α (Cell Signaling Technology, #4814, RRID: AB_390781, 1:1000), 3A (monoclonal antibody, prepared by our laboratory, 1:1000), α-syn (Abcam, #ab138501, RRID: AB_2537217, 1:1000), pS129 α-syn (Cell Signaling Technology, #23706, RRID: AB_2798868, 1:1000), tyrosine hydroxylase (TH) (Sigma, #AB152, 1:1000), Iba-1 (GeneTex, #GTX10042, RRID: AB_1240434, 1:1000), GFAP (Cell Signaling Technology, #3670S, RRID: AB_561049, 1:1000), Cleaved caspase3 (Beyotime, #AC033, 1:1000), interleukin-1β (IL-1β) (Beyotime, #AG2258, 1:1000), inducible nitric oxide synthase (iNOS) (Beyotime, #AF7281, 1:1000), sodium potassium ATPase (Beyotime, #AF1864, 1:1000), and β-actin (Abcam, #ab8266, 1:1000).

### Pull-down assay

Active RhoA and active Cdc42/Rac1 were pulled down by GST-Rok (Active Rho-binding domain, RBD) and GST-Pak (PAK1-binding domain, PBD) beads according to the previously reported method [22]. Briefly, the RBD and PBD fusion proteins were expressed in *Escherichia coli* strain BL21 (DE3) bacteria and captured on Glutathione Sepharose 4B (Cytiva, Marlborough, MA, #17075601). Protein lysates of CAR-A, ns-CAR modified astrocytes (ns-CAR-A) and negative control astrocytes transfected with empty vector (NC-A) treated with or without 1 μmol/L α-synOs for 24 h were mixed with 20 μL of the PBD or RBD beads and incubated at 4 °C for 45 min. Then, the beads were washed and the proteins bound on the beads were analyzed by Western blotting.

### ELISA

The levels of pro-inflammatory (IL-1β, tumor necrosis factor-α [TNF-α], interferon-γ [IFN-γ], IL-6) and anti-inflammatory cytokines (transforming growth factor-β [TGF-β], IL-4) in brain lysates of mice and cell culture supernatants were determined using corresponding ELISA kits (Biolegend) according to the manufacturer’s protocols. The levels of dopamine, 3, 4-dihydroxyphenylacetic acid (DOPAC) and homovanillic acid (HVA) in brain lysates of mice were determined using corresponding ELISA kits (Signalway Antibody, Shanghai, China). To test the affinity of 3A and α-synOs, 1 μg 3A was coated onto a 96-well plate at 4 °C overnight. The wells were blocked by 3% BSA in PBS. After washing with 0.1% PBST, serial diluted α-synOs were added to the wells and incubated for an hour at 37 °C. Bound α-synOs were detected by α-syn antibody (Abcam, #ab138501, RRID: AB_2537217) followed by secondary antibody labeled with HRP and 3,3′,5,5′-tetramethylbenzidine (TMB). The absorbance at 450 nm was measured using a SpectraMax M5 microplate reader.

### Quantitative RT-PCR analysis

RNA was extracted from cell lysates and brain homogenates using the RNeasy Lipid Tissue kit (Qiagen, Hilden, North Rhine-Westphalia, Germany, #74804) according to the manufacturer’s instructions. cDNA was prepared from total RNA using the PrimeScript RT-PCR kit (Takara, Kusatsu, Shiga, Japan, #RR037Q). Relative gene expression of the cDNA was assayed using a 7500 Fast real-time PCR instrument (Applied Biosystems) with SYBR Select Master Mix (Applied Biosystems, #4472908). RT-PCR data were analyzed by the ΔΔCT method by normalizing the expression of each gene to the housekeeping gene GAPDH and then to the control groups. The following primers were used: *Tnf-α*: 5′-GATTATGGCTCAGGGTCCAA-3′, 5′-GCTCCAGTGAATTCGGAAAG-3′; *Il-6*: 5′-CCGGAGAGGAGACTTCACAG-3′, 5′-TTGCCATTGCACAACTCTTT-3′; *Gapdh*: 5′-TGAATACGGCTACAGCAACA-3′, 5′-AGGCCCCTCCTGTTATTATG-3′; *Gpc4*: 5′-TGGAAGCGGATGTGAATATCAGCAG-3′, 5′-CAGAGAGCAGCGAGGAGGTAGG-3′; *Gpc6*: 5′-GCAGATCATGGCTCTCCGTGTG-3′, 5′-ACTGGATTCATCGCTTGTGTCTTGG-3′; *Thbs1*: 5′-ATGCCTGCGATGATGACGATGAC-3′, 5′-CTGGGCTGGGTTGTAATGGAATGG-3′; *Thbs2*: 5′-GCGATGCCTGCGACTCTGATG-3′, 5′-GTCTTCCTGGTCTGGGTTGAACAC-3′.

### RNA-seq analysis

Total RNA was isolated from astrocytes of mouse brain using an RNeasy mini kit (Qiagen). For each sample, 1 μg of total RNA was used for library preparation. The poly (A) mRNA isolation was performed using Oligo (dT) beads. mRNA fragmentation was performed using divalent cations under high temperature. Priming was performed using Random Primers. First-strand cDNA and the second-strand cDNA were synthesized. The purified double-stranded cDNA then underwent end repair and dA-tailing in one reaction, followed by a T-A ligation to add adaptors to both ends. Size selection of adaptor-ligated DNA was then performed using DNA Clean Beads. Each sample was then amplified by PCR using P5 and P7 primers and the PCR products were validated.

Then libraries with different indexs were multiplexed and loaded on an Illumina HiSeq/ Illumina Novaseq/MGI2000 instrument for sequencing using a 2 × 150 paired-end (PE) configuration according to the manufacturer’s instructions.

Differential expression analysis was carried out with the DESeq2 Bioconductor package (RRID: SCR_015687), a model based on negative binomial distribution. The estimates of dispersion and logarithmic fold changes incorporated data-driven prior distributions. *P*_adj_ ≤ 0.05 was set to identify genes with differential expression.

Kyoto Encyclopedia of Genes and Genomes (KEGG) is a collection of databases dealing with genomes, biological pathways, diseases, drugs, and chemical substances (RRID: SCR_012773). We used scripts in house to enrich significant differential expression gene in KEGG pathways.

### Animals

Four-month-old male C57BL/6 mice and A53T α-syn transgenic line M83 (B6; C3-Tg (Prnp-SNCA*A53T)83Vle/J, RRID: IMSR_JAX:004479) were purchased from Jackson Laboratory (Bar Harbor, ME). In this study, all animal experiments were performed in accordance with the China Public Health Service Guide for the Care and Use of Laboratory Animals. Experiments involving mice and protocols were approved by the Institutional Animal Care and Use Committee of Tsinghua University (15-LRT1). Mice were maintained at room temperature (~ 18–23 °C) with 40%–60% humidity. Mice were kept on a 14 h: 10 h light/dark cycle with free access to food and water.

### Mouse stereotaxic injection

A53T mice were injected with 10 μg of α-syn fibrils dissolved in 5 μL of PBS (1.25 μL per injection site) in the somatosensory cortex region (mediolateral, 2.0 mm from bregma; anteroposterior, 0.2 mm; dorsoventral, 0.8 mm) and the dorsal neostriatum region (mediolateral, 2.0 mm from bregma; anteroposterior, 0.2 mm; dorsoventral, 2.6 mm) in both hemispheres. C57BL/6 mice were injected with only PBS in both hemispheres. Three weeks after PFF injection, pole test was performed to verify the establishment of PFF-seeded A53T mouse model. Then, 4 μL (5 × 10^12^ vg/mL, 1 μL per injection site) of adeno-associated virus (AAV) expressing CAR or ns-CAR was injected into the somatosensory cortex (mediolateral, 2.0 mm from bregma; anteroposterior, 0.2 mm; dorsoventral, 0.8 mm) and the dorsal neostriatum (mediolateral, 2.0 mm from bregma; anteroposterior, 0.2 mm; dorsoventral, 2.6 mm). Control animals were injected with only PBS. The solution was injected at a rate of 0.2 μL/min and the needle was kept in place for another 5 min after injection for proper diffusion of the solution. The surgical site was cleaned with sterile saline and the incision sutured. After surgery, animals were monitored and post-surgical care was provided. The mice were trained and tested for their behavioral and cognitive abilities five weeks after the stereotaxic injection. Then they were sacrificed for biochemical and histological analysis after behavioral tests.

### Y-maze test

Y-maze test was performed to evaluate the spatial recognition memory of animals. The test consisted of 2 trials with an interval of 1 h. In the first trial, each mouse was allowed to explore 2 arms (the start and familiar arms) of the maze for 10 min, while the third arm (novel arm) was blocked. In the second trial, the mouse was put back to the same starting arm as in the first trial with free access to all three arms for 5 min. By using a ceiling-mounted charge-coupled device camera, all trials were recorded on a videocassette recorder, and the number of entries and the time spent in each arm were analyzed.

### Open field test

In the open field test, each mouse was placed in the central zone of a 40 × 40 cm square apparatus and allowed to move freely for 10 min. By using a ceiling-mounted charge-coupled device camera, all trials were recorded on a videocassette recorder. The time spent in the central zone and the distance travelled in the edge zone were calculated. The rearing frequency and defecation number of animals were also recorded.

### Pole test

Mice were initially habituated and trained 1 day prior to testing. Each mouse was placed on the top of a rough-surfaced wooden pole (50 cm in length and 1 cm in diameter) and allowed to descend to the base of the pole. During the test, each mouse was placed with its head oriented toward the top of the pole. The times required to turn the head downward and to descend the entire length of the pole were measured. Each test was performed with three trials with 1-h intervals. The performance of each mouse was expressed as the average of the three trials.

### Wire hang test

The wire hang test was designed to assess motor function. Each mouse was placed on the metal wire cage top. The cage top was then inverted and placed about 50 cm above the surface of the soft bedding in the home cage, and was manually shaken at a constant speed (4 times per second) in the air. The latency to fall from the cage top was recorded. Each test was performed with three trials with 1-h intervals. The performance of each mouse was expressed as the average of the three trials.

### Tissue lysate preparation

Tissues were homogenized in brain lysis buffer (containing 10 mmol/L Tris–HCl, pH 7.4, 150 mmol/L NaCl, 5 mmol/L EDTA, 1% Triton X-100, protease inhibitor cocktails and phosphatase inhibitor cocktails). The homogenate was centrifuged at 17,500× *g* for 20 min at 4 ℃. The supernatant was the detergent-soluble fraction of the tissue. The pellet was washed once by brain lysis buffer and homogenized in brain lysis buffer containing 1% SDS and 0.5% sodium deoxycholate. The homogenate was centrifuged and the resulting supernatant (Triton X-100-insoluble fraction) was collected.

### Immunohistochemistry (IHC) analysis

Mice were deeply anaesthetized by intraperitoneal injection of a mixture of ketamine (100 mg/kg) and xylazine (10 mg/kg) and immediately perfused with ice-cold PBS containing heparin (10 U/mL) and sacrificed. Mouse brains were immediately removed and divided along the sagittal plane. The left brain hemisphere was fixed in 4% PFA at 4 °C overnight, and cut into 5-μm sagittal sections using a Lecia CM1850 microtome. Before staining, the sections were incubated with sodium citrate buffer (10 mmol/L sodium citrate, pH 6.0) for 20 min at 95 °C for antigen retrieval. The sections were then permeabilized and blocked with 10% normal donkey serum in 0.3% Triton X-100 PBST for 1 h at room temperature and then incubated with primary antibodies, followed by corresponding fluorescently conjugated secondary antibodies (Abcam, Cambridge, UK). The sections were imaged on a Leica TCS SP8 confocal microscope. For 3’-diaminobenzidine (DAB) immunostaining, the sections were incubated with a primary antibody, followed by a corresponding HRP-labeled secondary antibody (Zsbio, Beijing, China, Cat. #zb230, #zb2301) and visualized with an Olympus IX73 inverted microscope with DP80 camera. The following primary antibodies were used: anti-3A (monoclonal antibody, prepared by our laboratory, 1:100), pS129 α-syn (Cell Signaling Technology, #23706S, RRID: AB_2798868, 1:100), TH (Sigma, #AB152, 1:1000), GFP (Abcam, #ab5450, RRID: AB_304897, 1:100), MAP2 (Abcam, #ab32454, RRID: AB_776174, 1:100), Iba-1 (GeneTex, #GTX100042, RRID: AB_1240434, 1:100), and GFAP (Cell Signaling Technology, #3670S, RRID: AB_561049, 1:100; Affinity, #DF6040, AB_2838012, 1:100; Abcam, #ab302644, 1:100). For Nissl staining, tissue sections were stained with Nissl Stain Kit (Solarbio, #G1434) according to the manufacturer’s protocols. The sections were then visualized with an Olympus IX73 inverted microscope with a DP80 camera. All images were analyzed by Image J Software 1.52a (NIH, MA).

### Statistics

Data were analyzed with GraphPad Prism v.8.0.1 (RRID: SCR_002798). Method of statistical test was denoted in each figure legend. All data are presented as mean ± SEM unless otherwise indicated. Data normal distribution was tested using the Shapiro–Wilk test. For normally distributed data, one-way or two-way ANOVA followed by Tukey’s test was used for comparisons among three or more groups, or an unpaired *t* test with two-tailed *P* values was used for two group comparisons. For data that were not normally distributed, Mann–Whitney test (two groups) or Kruskal–Wallis one-way ANOVA on ranks (three or more groups) with Two-stage step-up Benjamini, Krieger and Yekutieli test was used. *P* < 0.05 was considered as statistically significant.

## Results

### CAR is expressed on astrocytes

**Fig. 1:**
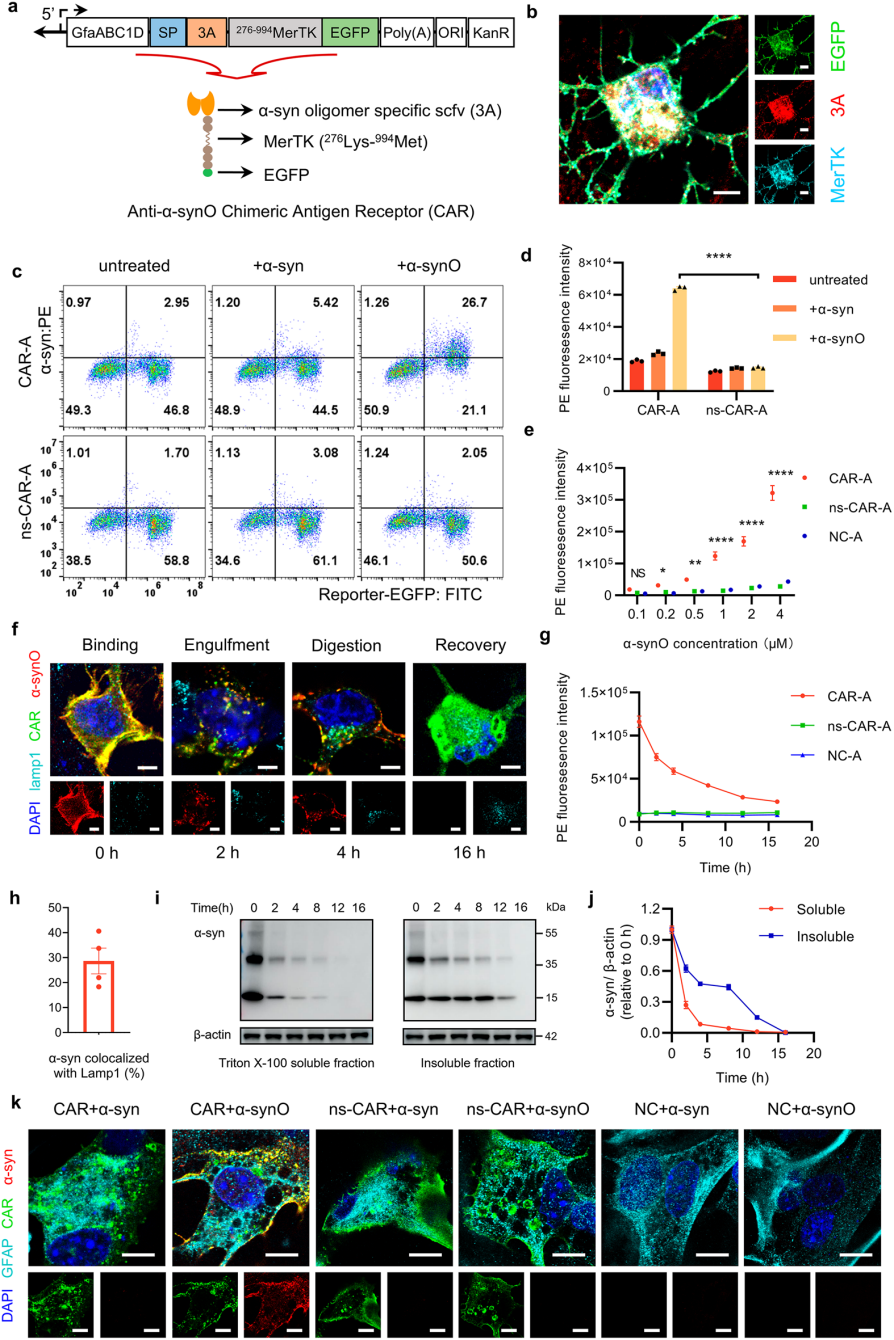
Expression of CAR-A and effect on α-synO phagocytosis and digestion. **a** Design of the CAR-expression plasmid. CAR was expressed in fusion with 3A scFv and enhanced green fluorescent protein (EGFP) under the control of the astrocyte-specific promotor GfaABC1D. SP, signal peptide; Poly(A), polyadenylation signal; ORI, origin of replication; KanR, kanamycin resistance gene. **b** Representative image of CAR expression on an astrocyte. The co-localization of 3A, MerTK and EGFP was assessed by confocal microscopy. Scale bars, 10 μm. **c** Flow cytometry analysis of the binding of CAR-A and ns-CAR-A to α-syn monomers and oligomers (α-synOs). The astrocytes were transfected with CAR or ns-CAR lipoplexes for 48 h. After 2 h-incubation with 1 μmol/L α-syn monomers (α-syn) or α-synOs, cells were stained with PE-labeled anti-α-syn antibody. **d** PE fluorescence in EGFP-positive astrocytes. *n* = 3 independent experiments. **e** Flow cytometry analysis of the amount of α-synO engulfed by CAR-A, ns-CAR-A and NC-A in the presence of different α-synO concentrations. *n* = 3 independent experiments. **f** Representative images depicting the phases of engulfment and digestion of α-synO by CAR-A. CAR-A was treated with 1 μmol/L α-synO, and the medium was changed after 1 h incubation. α-SynO and Lamp1 in CAR-A were stained with respective antibodies at different time points and imaged by confocal microscopy. Scale bars, 5 μm. **g** The kinetic curves of α-synO digestion in CAR-A, ns-CAR-A and NC-A. *n* = 3 independent experiments. **h** Statistical analysis of the proportion of α-syn colocalized with Lamp1 in digestion stage in (**f**) by Image J. *n* = 4 independent experiments. **i** Intracellular α-syn in Triton X-100-soluble and -insoluble fraction detected by Western blotting at different time points post astrocytic phagocytose of α-synOs. β-actin was used as a control. **j** Quantification of α-syn (**i**) using Image J. *n* = 3 independent experiments. **k** Representative images depicting the binding of ns-CAR-A, NC-A and CAR-A to α-syn monomers and oligomers. Scale bars, 5 μm. Data are mean ± S.E.M. One-way ANOVA (**d**) or Two-way ANOVA (**e**) followed by Tukey’s multiple comparison test was used for statistical analysis. *P < 0.05, **P < 0.01, ****P < 0.0001 indicate significance compared to respective groups

We constructed the anti-α-synO CAR by replacing the ligand-binding domain (19Gly-275Asn, Ig-like domain) of MerTK with 3A scFv. 3A is an scFv developed by our laboratory that can specifically bind to α-synOs but not monomers or fibrils (Fig. S1a, b). To verify the binding of 3A to α-synOs in complex biological matrices, we carried out immunoprecipitation to isolate α-synOs from brain lysates of PD transgenic mice using 3A, and then detected the α-synOs by western blot using an anti-α-syn monomer antibody. The results showed that 3A still bound to α-synOs in mouse brain lysates (Fig. S1c). To achieve specific expression of anti-α-synO CAR on astrocytes, we inserted the astrocyte-specific promoter GfaABC1D near the 3A gene. A signal peptide sequence was inserted before 3A for membrane targeting. EGFP was co-expressed at the carboxyl terminus of CAR to facilitate the detection (Fig. 1a). We also designed a non-specific CAR (ns-CAR) as a control by replacing 3A with a nonspecific scFv gene sequence. Next, plasmids were transfected into mouse primary astrocytes to examine the expression of CAR. We found that CAR was intactly expressed on the membrane of astrocytes and partially present in the cytoplasm (Fig. 1b), but not in neurons or microglia under the control of the GfaABC1D promoter in the plasmid (Fig. S1d, e).

We characterized the subpopulation of astrocytes with CAR expression using antibodies for A1 biomarker C3 and A2 biomarker S100a10, respectively. The results demonstrated that the A2-type astrocytes were more readily to express CAR (Fig. S1f). The expression of CAR was further verified by western blotting. The band of 3A was located at the similar molecular weight as that of CAR, indicating the intact expression of CAR on astrocytes (Fig. S1g). The transfection efficiency of CAR was about 41% in vitro (Fig. S1e). To examine the membrane targeting efficiency of CAR, we extracted membrane and cytosolic proteins from astrocytes transfected with CAR, ns-CAR, MerTK plasmid or NC. Then, the distribution of receptors on the cell membrane and cytoplasm was compared. The membrane targeting efficiency of the engineered CAR was not significantly different from that of MerTK (Fig. S1h, i).

### α-synO engulfment and degradation induced by CAR-A

Then, we evaluated the phagocytic and digestive functions of CAR-A on α-synOs. To obtain oligomeric α-syn, α-syn monomer was incubated at 37 °C with continuous shaking, and the aggregation state of α-synOs was determined by ThT fluorescence assay, TEM and western blotting (Fig. S1j–l). Primary astrocytes were transfected with CAR or control plasmids and then α-synOs were added to different groups of astrocytes. The flow cytometry results showed that CAR-A exhibited significantly enhanced binding to α-synOs but not to monomers (Fig. 1c, d), and this binding was significantly enhanced with increased α-synOs concentrations, while ns-CAR-A and NC-A did not show binding to α-synOs (Fig. 1e). Immunocytochemistry results indicated that the process of engulfment and digestion of α-synOs by CAR-A could be divided into four sequential steps (Fig. 1f): (i) binding, during which α-synOs interacted with CAR expressed on astrocytes; (ii) engulfment, during which α-synOs and CAR complexes were engulfed by astrocytes to form phagocytic vesicles; (iii) digestion, during which α-synOs were partially colocalized with the lysosomes, indicating that α-synOs were digested via the lysosomal pathway (Fig. 1h); and (iv) recovery, during which CAR-A returned to its previous condition. Correspondingly, the amount of α-synOs in the astrocytes was also significantly decreased over time (Fig. 1g). The phagocytosis and clearance of α-synOs by CAR-A were further examined by western blot. Consistent with the results of immunocytochemistry, α-syn levels in both Triton X-100-soluble and -insoluble fractions in CAR-A were significantly reduced within 16 h (Fig. 1i, j). Unlike the strong affinity of CAR for α-synOs in our immunofluorescence experiments, under consistent laser intensity, the binding of ns-CAR-A and NC-A to α-synOs was hardly observable (Fig. 1k).

### Phagocytic mechanism and anti-inflammatory effect of CAR-A

**Fig. 2:**
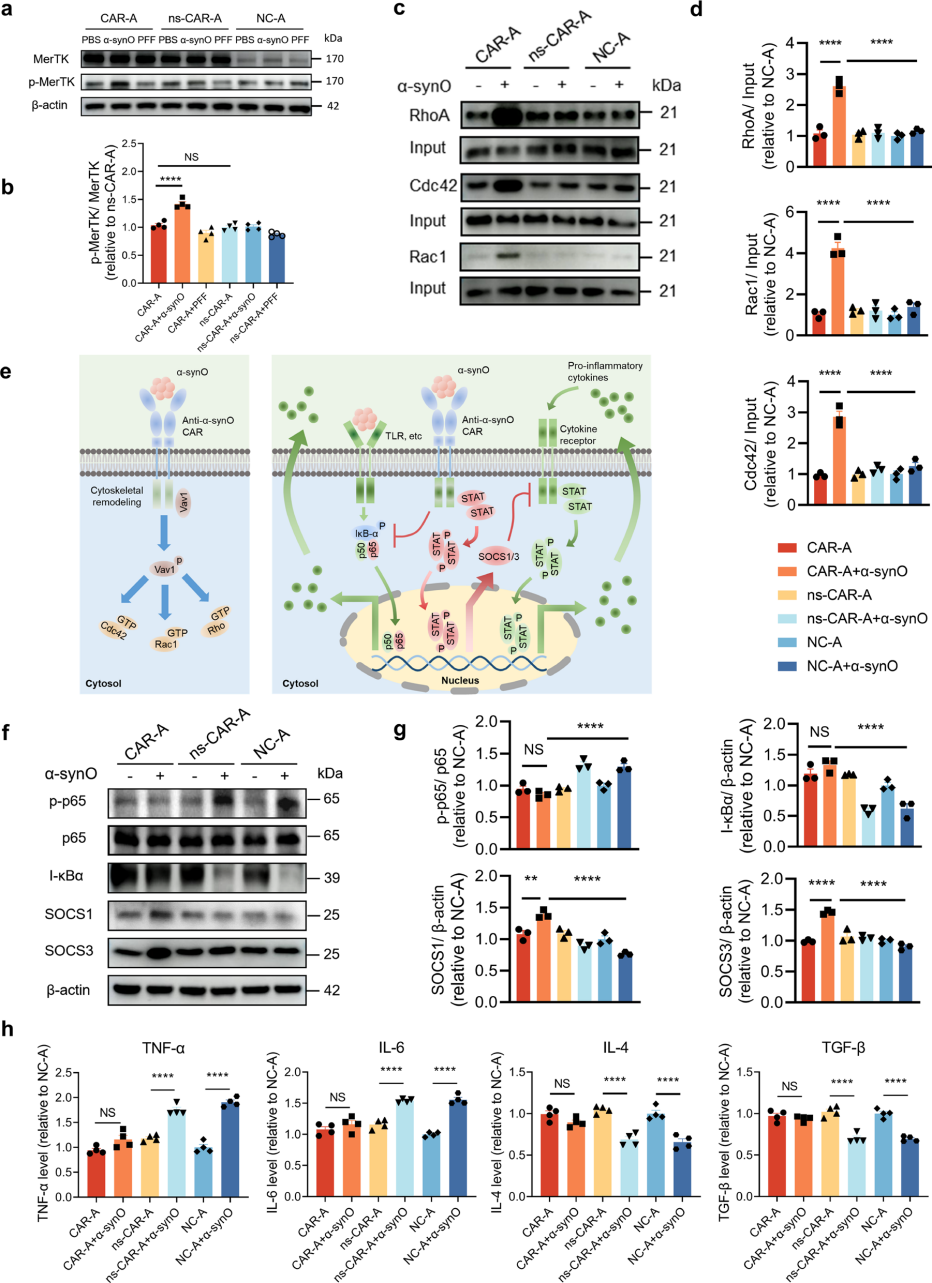
The phagocytic mechanism and anti-inflammatory effect of CAR-A. **a, b** Phosphorylation of MerTK in CAR-A, ns-CAR-A and NC-A treated with or without α-synOs for 24 h. The phosphorylation of MerTK was detected by western blotting (**a**). Levels of p-MerTK/MerTK quantified using Image J software, and normalized to that of ns-CAR-A in the absence of α-synOs (**b**). *n* = 4 independent experiments. **c, d** The activation of Rac1, Cdc42, and RhoA. Rac1, Cdc42, and RhoA were obtained from the lysates of CAR-A and other control astrocytes treated with or without α-synOs using GST-pull down assay, and analyzed by western blotting (**c**). Total Rac1, Cdc42, and RhoA were used as controls. The levels of Rac1, RhoA, and Cdc42 in (**c**) were quantified using Image J and normalized to that of the NC-A in the absence of α-synOs (**d**). *n* = 3 independent experiments. **e** Schematic representation of signaling pathways involved in α-synO phagocytosis and inhibition of pro-inflammatory cytokine release by CAR-A. **f** The protein levels of SOCS1, SOCS3, IκB-α, p65, and p-p65 in the cytoplasm of CAR-A and other control astrocytes treated with or without α-synO for 48 h. β-Actin was used as the control. **g** Relative levels of SOCS1, SOCS3, IκB-α and p-p65 were quantified using Image J and normalized to that of the NC-A in the absence of α-synO. *n* = 3 independent experiments. **h** The levels of pro-inflammatory cytokines (TNF-α, IL-6) and anti-inflammatory cytokines (IL-4, TGF-β) in CAR-A and other control astrocytes treated with or without α-synOs were determined by ELISA. *n* = 4 independent experiments. Data are mean ± SEM. One-way ANOVA followed by Tukey’s multiple comparison tests was conducted for statistical analyses (**b**, **d**, **g**, **h**). **P < 0.01, ****P < 0.0001 indicate significance compared to respective groups. NS, not significant

We then explored the signaling pathways involved in α-synO phagocytosis by CAR-A. A previous study showed that when two adjacent MerTK receptors simultaneously bind to the ligand, they will dimerize, followed by phosphorylation of the kinase domain and activation of the downstream signaling pathway [14]. We first evaluated the phosphorylation of CAR by western blotting analysis and found that CAR was activated and phosphorylated when it interacted with α-synOs, while α-syn PFFs did not activate CAR (Fig. 2a, b). Then, the activation of downstream Rho-family GTPases was verified by pull-down experiment, and the results showed that Rac1, Cdc42 and RhoA were activated in CAR-A but not in ns-CAR-A or NC-A during α-synO phagocytosis (Fig. 2c, d). This suggests that assembly of contractile actomyosin filaments and actin polymerization are involved in the phagocytosis of α-synOs by CAR-A [23]. Thus, binding of α-synOs to CAR on astrocytes leads to CAR phosphorylation, cytoskeletal remodeling, and α-synO phagocytosis (Fig. 2e). To investigate whether the C-terminal fusion expression of EGFP interferes with CAR activation, we compared the levels of phosphorylated Stat1 (p-Stat1), a downstream factor of MerTK activation [24], in CAR-A with or without EGFP fusion (CAR/E) when interacting with α-synOs. The results showed that fusion expression of EGFP at the C-terminus of CAR did not affect CAR activation (Fig. S1m, n).

Previous studies have shown that α-synOs could interact with Toll-like receptor 2 on astrocytes and activate the NF-κB pathway, thereby decreasing the level of κBα degradation inhibitor (IκB-α) and increasing the level of phosphorylation of p65 (p-p65), and ultimately lead to upregulation of pro-inflammatory cytokines in astrocytes [25, 26]. Moreover, MerTK activation inhibited the NF-κB pathway and the cytokine-receptor cascade (Fig. 2e) [24, 27]. We here detected the effect of CAR-A on inflammatory pathways using western blotting. Consistent with previous reports, α-synOs activated the NF-κB pathway in ns-CAR-A and NC-A, indicated by the decreased levels of IκB-α and increased level of p-p65. However, the binding of α-synOs to CAR-A dumbed the NF-κB pathway, and the levels of IκB-α and p-p65 did not change significantly (Fig. 2f, g). Furthermore, the binding of α-synOs to CAR-A enhanced the expression of SOCS1/3 (Fig. 2f, g), thereby inhibiting the feed-forward expansion of cytokines by reducing the release of pro-inflammatory cytokines, including TNF-α and IL-6, and preventing the decrease of anti-inflammatory cytokines, including IL-4 and TGF-β (Fig. 2h). Consistently, CAR activation prevented the increase of transcription of pro-inflammatory cytokines TNF-α and IL-6 in astrocytes after α-synO treatment (Fig. S2a, b).

Changes in the brain microenvironment are crucial for the pathogenesis of PD. Reactive astrocytes under pathological conditions lose their supporting role for neurons and release a variety of toxic chemokines and cytokines, leading to neuronal death and pro-inflammatory microglial activation [6, 28]. To examine the effects of CAR-A on microglia and neurons during α-synO clearance, conditioned medium (CM) from CAR-A and other experimental cells was collected and added to the medium of microglial and neuronal cultures (Fig. S2c). Compared with the medium from ns-CAR-A and NC-A, CM from CAR-A treated with α-synOs did not induce pro-inflammatory cytokine production in cultured microglia (Fig. S2d–f), and maintained the length of axons and dendrites without causing neuronal damage (Fig. S2g–i). In addition, TUNEL assay results indicated that CM from ns-CAR-A and NC-A rather than from CAR-A treated with α-synOs caused significant neuronal apoptosis (Fig S2j, k).

### CAR-A retains major astrocytic biological function

To investigate the effect of CAR on astrocytic function and metabolism, we used RNA-sequencing analysis to measure gene expression files in CAR-A and NC-A. Astrocyte purity was confirmed by RNA-seq data (Fig. S3a). RNA-seq analysis indicated that genes implicated in complement and coagulation cascades (Fga, Fgb, Serpina1 and Serpina3 gene families) were downregulated in CAR-A (Fig. S3b). KEGG pathway analysis showed that complement and coagulation cascades pathways were significantly enriched (Fig. S3c). It is noteworthy that the increased expression of these genes has been observed in the brains of A53T mice compared with wild-type (WT) mice [29]. Serpina3n, a member of the serpina3 gene family, is upregulated in aging astrocytes [30]. Serpina1 has also been found to be upregulated in the cerebrospinal fluid of PD patients [31]. The downregulation of genes implicated in complement and coagulation cascades in CAR-A could reduce astrocyte activation and may have beneficial effects on neurons and glia.

We then investigated the effect of CAR transfection on the expression of genes encoding proteins involved in basic functions of astrocytes, including activation markers GFAP and Vim, water channel Aqp4, cytosolic markers Aldh1l1 and Aldoc, potassium uptake channel Kcjn10, glutamate-metabolizing enzyme glutamine synthetase (Glul), glutamate and aspartate transporter Slc1a3, γ-aminobutyric acid transporter Slc6a11, and the component of the lactate shuttle (Ldha) [30]. There was no significant difference in the expression of these genes between CAR-A and NC-A (Fig. S3d). Moreover, CAR expression had no significant effect on the expression of genes involved in cholesterol metabolism, such as *Srebf1, Acat2, Hmgca1*, which is essential for the physiological activities of neurons [32] (Fig. S3f). Previous reports demonstrated that astrocytes secrete glypican (Gpc4 and Gpc6), Sparcm, thrombospondin (Thbs1 and Thbs2) and TGF-β family to induce excitatory synapse formation [33–36], and that MEGF10 and MERTK play critical roles in the astrocyte-mediated synapse elimination [12]. The expression of these genes was not significantly affected by CAR expression, except of MerTK, since CAR was based on MerTK (Fig. S3e). RT-PCR results showed that the transcription levels of glypican and thrombospondin were not significantly different between CAR-A and NC-A (Fig. S3g). These results suggest that CAR-A retains major astrocytic biological functions.

### CAR-A alleviates motor and cognitive impairment in A53T mouse model

We next investigated the therapeutic effect of CAR-A in PD-like mouse model. To induce a parkinsonian mouse model, we firstly incubated α-syn monomers in vitro and monitored fibril formation by ThT fluorescence assay and western blotting (Fig. S1j, S4b). TEM was used to visualize the fibrillar structure of α-syn PFFs (Fig. S4c). We then added sonicated PFFs to α-syn monomers and found that sonicated α-syn PFFs accelerated monomer aggregation (Fig. S4d, e), which confirmed the pathogenicity of α-syn fibrils. Next, we injected PFFs to somatosensory cortex and dorsal neostriatum in both hemispheres of 4-month-old A53T transgenic mice to accelerate the pathological development of PD [37] (Fig. S4a, f). The motor ability of these A53T mice was evaluated by the pole test 3 weeks post PFF seeding. Compared with the WT mice, A53T mice receiving α-syn PFF injection (PFF-seeded A53T mice) showed remarkably impaired motor ability, which indicated the establishment of the parkinsonian model (Fig. S4g, h).

Next, the PFF-seeded A53T mice were injected with AAV expressing CAR or ns-CAR, or PBS, in the somatosensory cortex and dorsal neostriatum. We firstly examined the expression and distribution of CAR in the brain by observing the EGFP-expressing cells 5 weeks post-injection. CAR was effectively expressed in mouse dorsal neostriatum, substantia nigra and cortex (Fig. S4i), and was expressed in astrocytes, but not in neurons and microglia (Fig. S4j, k). Moreover, we verified CAR expression in the mouse striatum by western blotting. Results showed that the levels of MerTK and 3A were significantly increased in PFF-seeded A53T mice infected with AAV-CAR (Fig. S4l).

**Fig. 3:**
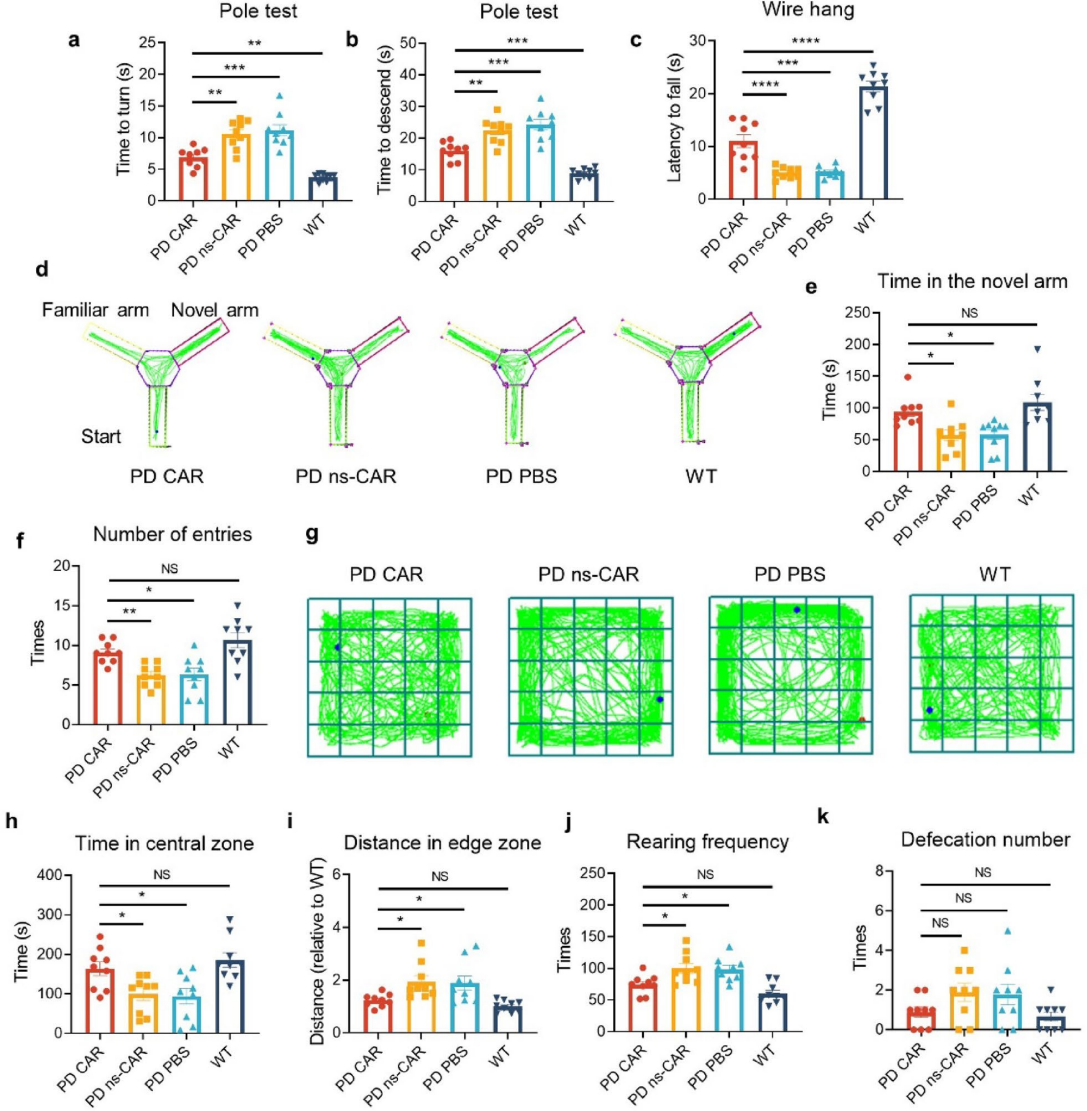
CAR-A attenuated motor and cognitive deficits in PFF-seeded A53T mice. PFF-seeded A53T mice were administered with PBS or AAV expressing CAR or ns-CAR via stereotaxic injection, and wild type (WT) mice were injected with PBS. The behavioral tests were carried out five weeks after injection. **a, b** The time for the mouse to turn on the pole and face downwards (**a**) and to descend from top to bottom of the pole (**b**) in the pole test. *n* = 9 biologically independent animals. **c** The latency of mice falling from the cages in wire hang test. *n* = 9 biologically independent animals. **d** Representative tracks in Y maze test for different experimental groups. **e, f** The time spent in the novel arm (**e**) and the number of entries in the new arm (**f**) in Y-maze test for different experimental groups. *n* = 9 biologically independent animals. **g** Representative tracks in the open field test in different experimental groups. **h–k** The time spent in the central zone (**h**), the distance travelled in the edge zone (**i**), rearing frequency (**j**) and defecation number (**k**) for different group mice in the open field. *n* = 9 biologically independent animals. Data are mean ± SEM. **a-c**, **e**, **h**, **i** One-way ANOVA followed by Tukey’s multiple comparison tests was conducted for statistical analyses. **f**, **j**, **k** Kruskal–Wallis one-way ANOVA with two-stage step-up Benjamini, Krieger, and Yekutieli test was used for statistical analysis. *P < 0.05, **P < 0.01, ***P < 0.001, ****P < 0.0001 indicate significance compared to respective groups. NS, not significant

The effect of CAR-A on the motor symptoms and the muscle strength of PFF-seeded A53T mice was evaluated by the pole test and wire hang test. The PFF-seeded 6-month-old A53T mice with PBS injection showed significant impairment in motor and cognition compared with the WT mice. In the pole test, CAR treatment significantly decreased the time to turn downward on the pole (time to turn) and to reach the bottom of the pole (time to descend) in PFF-seeded A53T compared with ns-CAR or PBS treatment (Fig. 3a, b). Consistently, the CAR-treated mice showed a significant increase in the latency to fall compared with the ns-CAR- or PBS-treated mice in the wire hang test (Fig. 3c).

In addition to motor symptoms, PD is associated with many non-motor symptoms, including cognitive impairment and anxiety [38]. We further evaluated the spatial memory and anxiety of mice by Y-maze test and open field test. In the Y-maze test, the PFF-seeded A53T mice treated with CAR spent more time in the novel arm and made more entries into this arm compared with ns-CAR- or PBS-treated mice (Fig. 3d–f). In the open field test, CAR-treated mice showed more time spent in the central zone, less distance travelled in the edge zone, and less rearing times compared with ns-CAR- or PBS-treated mice (Fig. 3g–j). The CAR-treated mice exhibited fewer defecation times, but there was no significant difference (Fig. 3k). These results indicate that the motor and cognitive deficits are relieved in CAR-treated PD-like mouse model.

### CAR-A reduces α-syn aggregate levels in A53T mouse brain

**Fig. 4:**
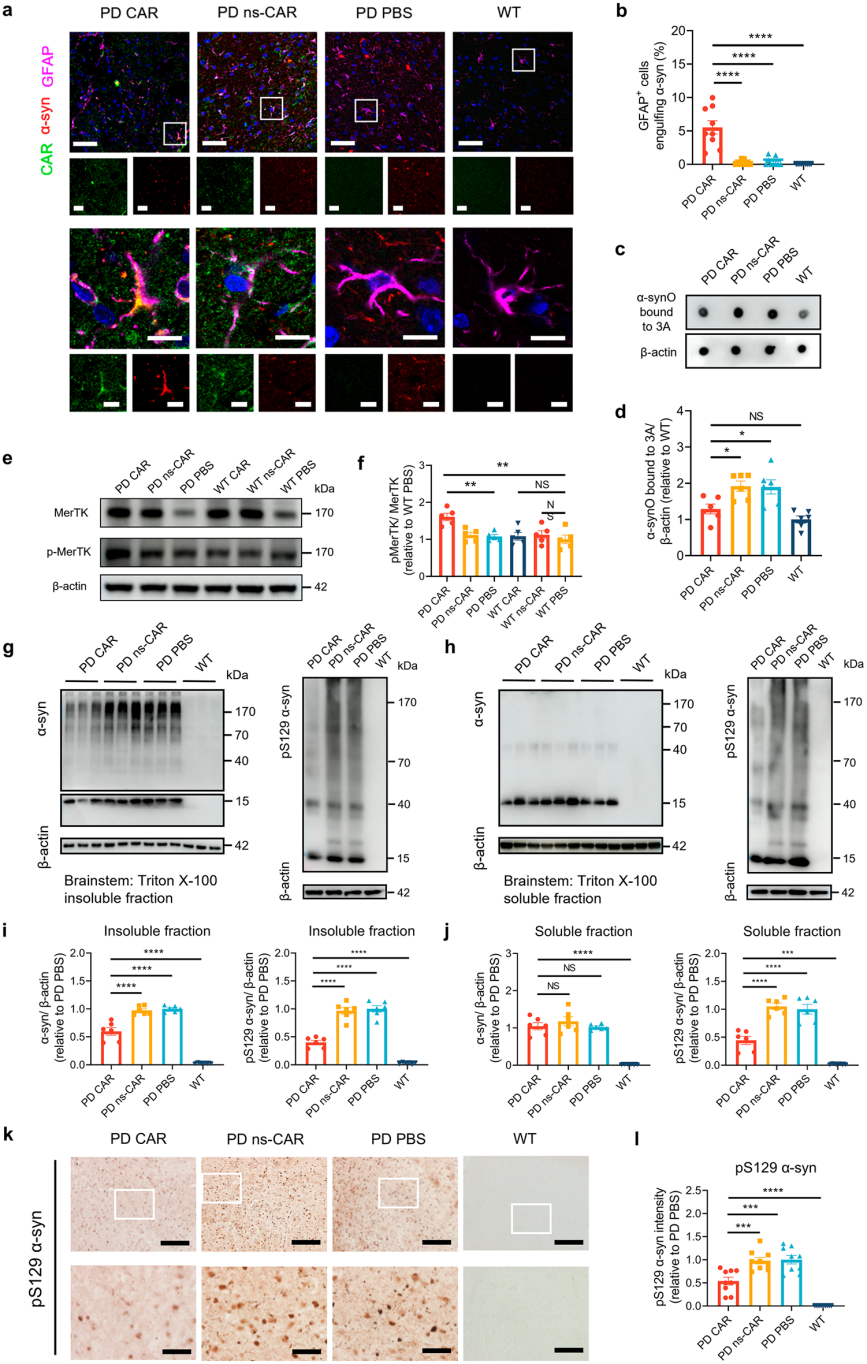
CAR-A reduced α-syn levels in PFF-seeded A53T mice. **a** Representative images depicting α-synO engulfment in different experimental groups in the striatum. Scale bars, 50 μm (upper four panels and eight small images below them) and 10 μm (lower four magnifications of zoomed areas and eight small images below them). **b** Quantification of the engulfed α-syn in GFAP-positive cells using Image J. *n* = 9 biologically independent animals. **c, d** The levels of α-synO specifically detected by 3A in brainstem were assessed by immunoblotting (**c**) and quantified by Image J (**d**). *n* = 6 biologically independent animals. **e** p-MerTK levels in the striatum of different groups assessed by western blotting. MerTK was used as a control. **f** Quantification of p-MerTK levels in (**e**) using Image J. *n* = 5 biologically independent animals. **g, h** Total α-syn levels and pS129 α-syn levels in Triton X-100-insoluble (**g**) and -soluble (**h**) fractions of brainstem assessed by western blotting. β-Actin was used as a control. **i, j** Quantification of total α-syn levels and pS129 α-syn levels in Triton X-100-insoluble (**i**) and -soluble (**j**) fractions of brainstem using Image J. *n* = 6 biologically independent animals. **k, l** The levels of pS129 α-syn in the substantia nigra were monitored by immunostaining (**k**), and quantified by Image J (**l**). Scale bar, 200 μm (low-magnification images) and 50 μm (high-magnification images), respectively. *n* = 9 biologically independent animals. Data are mean ± SEM. One-way ANOVA followed by Tukey’s multiple comparison tests was conducted for statistical analyses. *P < 0.05, **P < 0.01, ***P < 0.001, ****P < 0.0001 indicate significance compared to respective groups. NS, not significant

To evaluate the phagocytosis of α-synOs by CAR-A in vivo, we detected α-synOs in astrocytes in mouse striatum. The results demonstrated that AAV-CAR greatly enhanced α-syn engulfment by astrocytes compared to AAV-ns-CAR or PBS (Fig. 4a, b). In addition, the α-syn phagocytosed by astrocytes was digested through the lysosomal pathway (Fig. S4m). Consistently, the levels of 3A-recognized α-synOs were significantly decreased in the brainstem of A53T mice treated with CAR relative to ns-CAR and PBS (Fig. 4c, d). To examine whether CAR is specifically activated in PFF-seeded A53T mice, we injected AAV expressing CAR into the somatosensory cortex and dorsal neostriatum of both PFF-seeded A53T mice and WT mice. We found that the p-MerTK levels were significantly increased in the CAR-treated A53T mice, suggesting that CAR was significantly activated in A53T mice but not in WT mice (Fig. 4e, f). Moreover, CAR significantly decreased the α-syn levels in Triton-X100-insoluble fractions of brainstem in PFF-seeded A53T mice, compared to ns-CAR or PBS (Fig. 4g, i), while no significant change in α-syn level in the Triton-X100-soluble fraction was observed (Fig. 4h, j). Phosphorylation of α-syn at serine 129 (pS129) is generally associated with the formation of pathogenic α-syn aggregates. The levels of pS129 α-syn were markedly reduced in both Triton-X100-insoluble and -soluble fractions of brainstem in CAR-treated PFF-seeded A53T mice compared to that in ns-CAR or PBS-treated mice (Fig. 4g–j). Consistently, our IHC results also showed that CAR treatment significantly reduced pS129 α-syn levels in the substantia nigra (SN) of PFF-seeded A53T mice (Fig. 4k, l). We then evaluated α-syn levels in the cortex by western blotting and IHC assay. Consistent with the results in brainstem, CAR treatment significantly lowered α-syn levels in the Triton-X100-insoluble fraction but not in the soluble fraction (Fig. S5a–d), and significantly decreased the level of pS129 α-syn in the cortex of CAR-treated PFF-seeded A53T mice, compared to the ns-CAR or PBS group (Fig. S5e, f).

### CAR-A increases the levels of TH and neurotransmitters in the PD mouse model

**Fig. 5:**
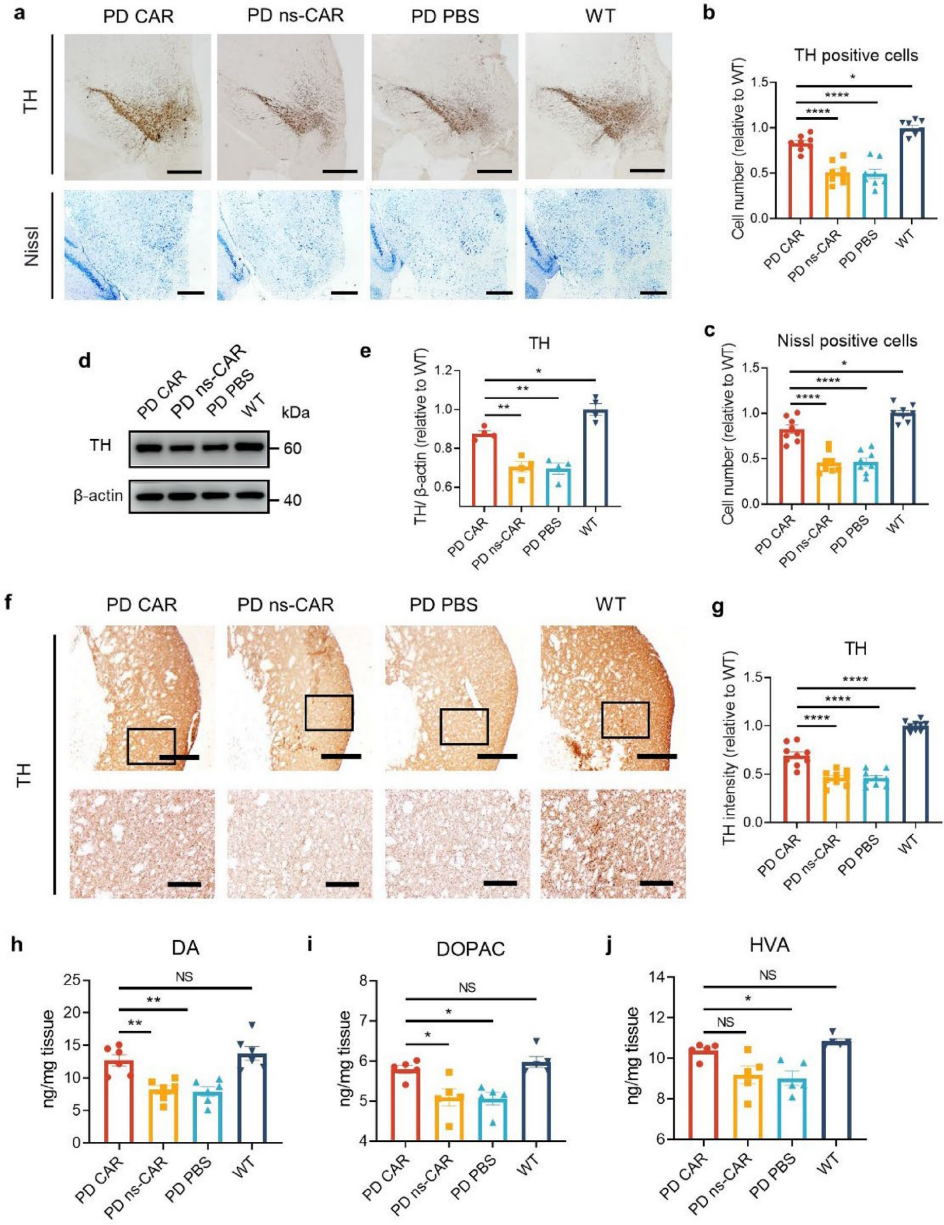
CAR increased TH levels in PFF-seeded A53T mice. **a** TH-positive cells and Nissl-positive cells in the substantia nigra (SN) of different group mice evaluated by IHC staining and Nissl staining. Scale bar, 200 μm. **b, c** Unbiased stereologic counts of TH-positive cells (**b**) and Nissl-positive cells (**c**) by Image J. *n* = 8 biologically independent animals. **d** TH protein levels in the SN monitored by western blotting. β-actin was used as a control. **e** Quantification of TH protein levels in (**d**) using Image J. *n* = 4 biologically independent animals. **f** TH neuronal fibers in the striatum detected by IHC staining. Scale bar, 400 μm (low-magnification images) and 100 μm (high-magnification images), respectively. **g** Relative TH fiber intensity in (**f**) quantified by Image J. *n* = 9 biologically independent animals. **h-j** The levels of dopamine (DA) (**h**), 3, 4-dihydroxyphenylacetic acid (DOPAC) (**i**), and homovanillic acid (HVA) (**j**) in striatum measured by ELISA. *n* = 5–6 biologically independent animals. Data are mean ± SEM. One-way ANOVA followed by Tukey’s multiple comparison tests was conducted for statistical analyses. *P < 0.05, **P < 0.01, ****P < 0.0001 indicate significance compared to respective groups. NS, not significant

α-SynOs induce dopaminergic neuronal loss in the SN and striatal dopamine deficiency, which are important pathological features of PD [38]. To evaluate the effect of CAR-A on dopaminergic neurons, we monitored the vulnerability of dopaminergic neurons in the SN by IHC and Nissl staining. The PFF-seeded A53T mice showed significantly decreased TH-positive neurons and Nissl-positive neurons in the SN compared to WT mice, while CAR but not ns-CAR treatment partially restored the numbers of these neurons (Fig. 5a–c). Compared with ns-CAR- or PBS-treated A53T mice, the level of cleaved caspase 3 was significantly reduced in the SN of CAR-treated mice, indicating that CAR ameliorated the apoptosis and the loss of TH-expressing cells in mouse brains (Fig. S5g, h). The level of TH in the SN was further confirmed by western blotting (Fig. 5d, e). Moreover, the TH fibers in the striatum were also significantly increased with CAR treatment (Fig. 5f, g). Since the recovery of TH-positive neurons in the SN and terminals of striatum leads to increased levels of dopamine and its metabolites DOPAC and HVA [39], we next tested these neurotransmitters in the striatum by ELISA. The levels of dopamine, DOPAC and HVA were decreased in the striatum of PFF-seeded A53T mice, while CAR treatment markedly ameliorated the loss of these neurotransmitters (Fig. 5h–j). These findings indicate that CAR treatment reduces the depletion of dopaminergic neurons in PFF-seeded A53T mice.

### CAR-A alleviates neuroinflammation in the PD mouse model

**Fig. 6:**
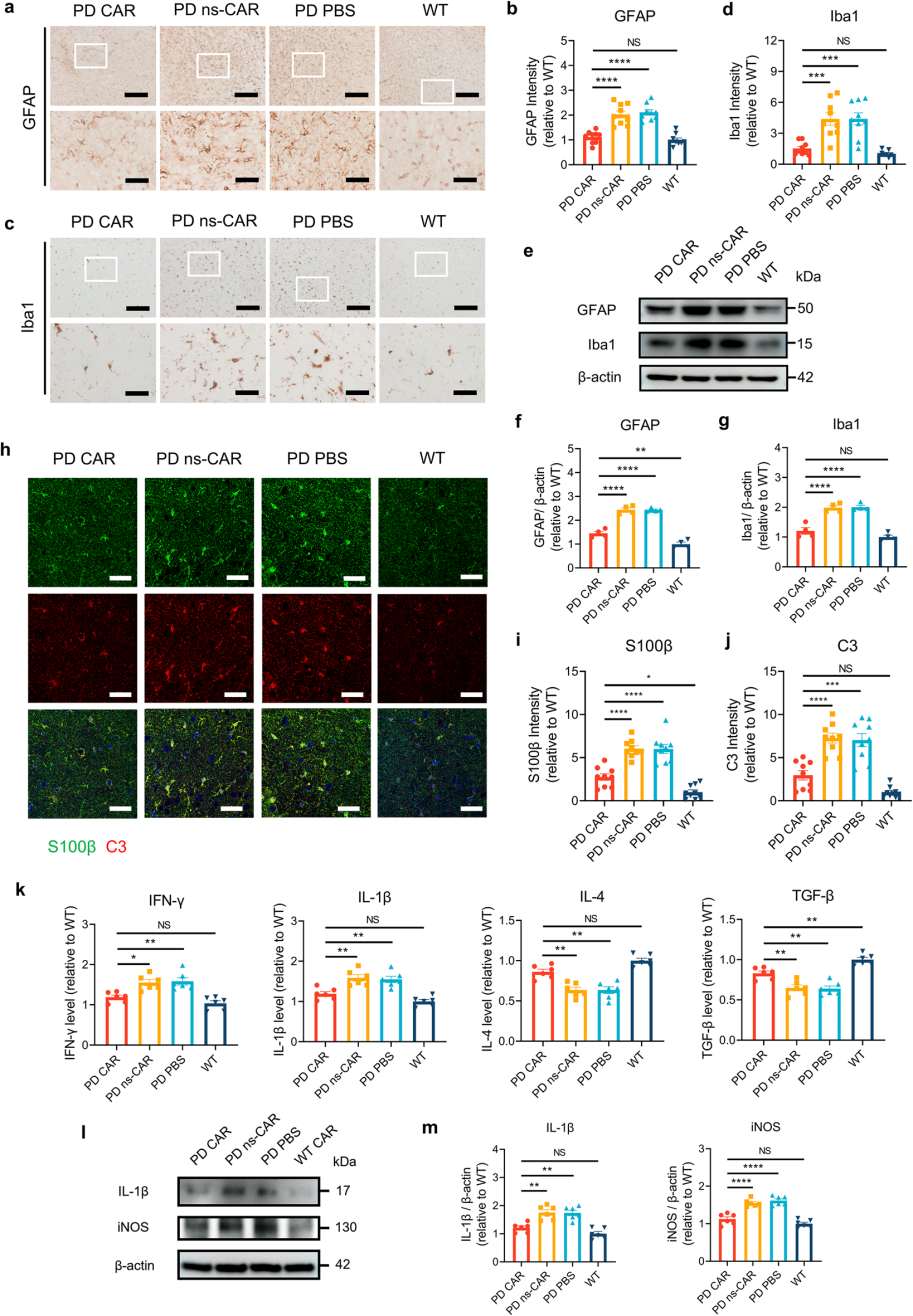
CAR-A reduced inflammatory response in the substantia nigra (SN) of PFF-seeded A53T mice. **a** Representative images of astrocytes in the SN of different group mice detected by anti-GFAP antibody. Scale bar, 200 μm (low-magnification images) and 50 μm (high-magnification images), respectively. **b** Levels of GFAP in (**a**) quantified by Image J. *n* = 9 biologically independent animals. **c** Representative images of microglia in the SN of different group mice detected by anti-Iba-1 antibody. Scale bar, 200 μm (low-magnification images) and 50 μm (high-magnification images), respectively. **d** Levels of Iba-1 quantified by Image J. *n* = 9 biologically independent animals. **e** The protein levels of GFAP and Iba-1 in SN measured by western blotting. β-Actin was used as a control. **f, g** Quantification of GFAP (**f**) and Iba1 (**g**) in (**e**) using Image J. *n* = 4 biologically independent animals. **h** The expression of S100β and C3 in the SN determined by IHC. **i, j** Quantification of S100β (**i**) and C3 (**j**) in (**h**) using Image J. *n* = 9 biologically independent animals. **k** The levels of pro-inflammatory cytokines (IFN-γ and IL-1β) and anti-inflammatory cytokines (IL-4 and TGF-β) in the SN of mice were determined using ELISA. *n* = 6 biologically independent animals. **l** The levels of pro-inflammatory cytokines IL-1β and iNOS in brainstem determined using western blotting. β-actin was used as a control. **m** Quantification of IL-1β and iNOS in (**l**) using Image J. *n* = 6 biologically independent animals. Data are mean ± SEM. One-way ANOVA followed by Tukey’s multiple comparison tests was conducted for statistical analyses. *P < 0.05, **P < 0.01, ***P < 0.001, ****P < 0.0001 indicate significance compared to respective groups

α-SynOs activate glial cells, inducing gliosis and production of neurotoxic pro-inflammatory cytokines, which in turn promotes dopaminergic neuron death, contributing to PD pathology. Injection of α-syn PFF to A53T mice significantly increased the numbers of GFAP-positive astrocytes and Iba-1-positive microglia in the SN and the striatum, while CAR rather than ns-CAR treatment significantly decreased microgliosis and astrogliosis (Fig. 6a–d and Fig. S5i–l). These results were further confirmed by western blotting analysis (Fig. 6e–g). The expression of C3 and S100β, markers of reactive astrocytes [40], was significantly reduced in the SN of CAR-treated mice, indicating that CAR prevented astrocyte activation in the brains of PFF-seeded A53T mice (Fig. 6h–j). Consistently, the expression of pro-inflammatory cytokines (IFN-γ and IL-1β) was significantly down-regulated and the expression of anti-inflammatory cytokines (IL-4 and TGF-β) was markedly increased in the SN of CAR-treated PFF-seeded A53T mice (Fig. 6k). Furthermore, the levels of pro-inflammatory cytokines (IL-1β and iNOS) in the brainstem were significantly decreased in the CAR-treated PFF-seeded A53T mice compared to the PBS- or ns-CAR-injected mice (Fig. 6l, m). These results indicate that CAR-A effectively attenuates neuroinflammation and improves the brain microenvironment in PFF-seeded A53T mice.

## Discussion

α-SynOs play a key role in the pathogenesis of PD by inducing neuron dysfunction and glial inflammatory responses. α-SynOs also have prion-like seeding and propagation activity, contributing to the spread of PD pathology [1]. Astrocytes are the most abundant cell type in the brain and play an important role in maintaining CNS and clearing cell debris and pathogens. However, under pathological conditions, astrocytes lose physiological function and convert to a reactive phenotype with low phagocytic function and release of inflammatory factors. This breaks the brain microenvironment, promotes α-synO spread and contributes to PD pathogensis [5, 7]. Therefore, endowing reactive astrocytes with the ability to engulf α-synOs while simultaneously blocking the generation of inflammatory factors would be an ideal strategy for PD treatment. We here successfully constructed and expressed α-synO-targeting CAR on astrocytes based on the MerTK receptor. The CAR showed beneficial effects in clearing α-synOs and inhibiting inflammatory factor generation in vitro and in vivo.

Compared with astrocytes, microglia are a type of macrophages resident in the CNS, and play more important roles in clearance with higher degradation efficiency. However, these microglial characteristics result in a much lower AAV transfection rate in microglia [41]. Given the high number of astrocytes in the CNS, we chose astrocytes to be engineered for CAR expression. As activation of many phagocytic receptors on glia induces the production of inflammatory factors [11], we chose MerTK, an anti-inflammatory phagocytic receptor, to construct CAR, in order to prevent inflammatory responses when the recombinant receptor is activated.

Previous studies have demonstrated that expression of genes in the complement and coagulation cascades is upregulated in astrocytes during aging and in PD patients [42]. The single-cell transcriptomic atlas for the α-syn-A53T PD mouse brain reveals elevation of NF-κB activity [43], which is consistent with our results in cultured cells. Many antibodies against α-syn have been developed for PD treatment. Although some showed therapeutic benefits in PD model animals, they all failed in clinical trials with inefficiency and side effects. This may be partially attributed to the disruption of the normal physiological function of the target protein by antibodies and the Fc receptor-mediated pro-inflammatory responses and non-specific immune cell activation triggered by antibodies [44, 45]. Therefore, to improve the therapeutic outcomes of PD treatment, accurate clearance of α-synOs while creating a non-inflammatory microenvironment is urgently needed. The CAR in the present study was designed by replacing the ligand-binding domain of MerTK with an α-synO-specific scFv, promoting the accuracy of pathogen targeting, preventing inflammatory responses induced by conventional antibodies, and overcoming the problem of short half-life of scFv antibodies. The activation of MerTK requires binding of two neighbouring receptors to the ligand. α-SynOs displaying multiple epitopes can readily bind to two CARs, activating MerTK, leading to activation of Rac1, Cdc42 and RhoA. This results in significantly enhanced phagocytosis of α-synOs, and decreased release of pro-inflammatory cytokines through inhibiting the NF-κB pathway and cytokine receptor signal pathway.

Numerous studies have applied PFFs as a seed to induce the aggregation of intracellular α-syn, and a few of formed α-syn aggregates then were excreted to extracellular space and spread in flowing intercellular fluid within the brain, resulting in PD occurrence and development [46]. The CAR receptors anchored on the cell membrane bind the α-synOs in flowing intercellular fluid and clear them. Thus, even CAR expression was limited to certain regions, and CAR-A number was not high, CAR receptors still effectively cleared α-synOs. In addition, although PFFs and CAR were injected into the striatum, our results revealed a reduction of α-syn aggregates in the substantia nigra of CAR-treated PD mice. These results indicate that CAR in the striatum can block the seeding and propagation of the endogenous α-syn aggregates.

In the brain, microglia are the most important player in clearance. However, it is very difficult to transfect microglia with plasmids, as microglia have a natural and strong intracellular degradation ability. Astrocytes have a certain phagocytic and clearance function, and plasmids are easy to be transformed and expressed in them. Astrocytes are the most abundant glial cells in the brain, and the concentration of soluble α-synOs responsible for pathological spread in the brain extracellular fluid is very low [47]. Even 5% of the astrocytes are enough to block the spread of α-synOs existing in the flowing intercellular fluid. In this study, we characterized the formation of α-synOs, and compared the distribution of astrocytes/astrogliosis in the tissues of PD model mice treated with PBS and WT mice. Consistent with previous reports [48], PFF-induced A53T PD model mice treated with PBS displayed high levels of α-synOs (Fig. 4g-j) and remarkable astrogliosis compared with WT controls (Fig. 6a,b). In addition, the construction of CAR did not affect the distribution and number of astrocytes in the mouse striatum compared to the PBS group (Fig. S5i).

In the PD-like mouse model, CAR-A was effectively activated, which ameliorated α-syn pathology, created a healthy microenvironment for neurons, restored dopaminergic neurons as well as TH and dopamine levels, and ameliorated motor and cognitive deficits. Compared with traditional drug treatment, engineered cell therapy offers spatiotemporal interventions in a homeostatic manner [49]. In this study, CAR was significantly activated in the presence of α-synOs, and the activity would return to a low state when proteostasis is re-established. Similarly, the anti-inflammatory effect of CAR would be limited by the appearance time and location of α-synOs. Unlike CAR-T cell therapy in the treatment of peripheral tumors, it is challenging to reinfuse a large number of engineered cells to the CNS. So far, therapies using induced pluripotent stem cells still face safety concerns [50], and cell transplantation may result in strong immune responses. Therefore, we engineered the cells in situ in brains to treat PD-like pathology and symptoms in the mouse model.

In summary, the in situ CAR-A-based strategy is an effective treatment for PD-like mouse model. Our study suggests that the novel therapeutic platform using the CAR that contains different scFv antibodies to eliminate toxic aggregates would have a promising future in the treatment of various neurodegenerative diseases. Moreover, in addition to our present CAR designed to exert a phagocytic function, other types of synthetic receptors that perform different or combined functions are warranted to be engineered to treat various diseases.

## Conclusions

We have developed a novel anti-inflammatory CAR designed to recognize α-synOs, achieving the dual purpose of clearing α-synOs and suppressing neuroinflammatory responses. CAR-A has demonstrated promising therapeutic effects in a PD-like mouse model.

## Supplementary Information

**Additional file 1**. **Fig. S1** The expression of CAR in vitro. **Fig. S2** The effect of CAR-A on the neurons and microglia in vitro.** Fig. S3** CAR expression did not interfere the physiological function of astrocytes. **Fig. S4** Establishment of PFF-seeded A53T mouse model and the expression of CAR in vivo. **Fig. S5** CAR decreased pathology in PFF-seeded A53T mice.

## Data Availability

All data generated and/or analyzed during this study are either included in this article or are available from the corresponding author on reasonable request.

## Abbreviations

α-syn: α-synuclein
CAR: Chimeric antigen receptor
Cdc42: Cell division cycle 42
EGFP: Enhanced green fluorescent protein
GFAP: Glial fibrillary acidic protein
Iba-1: Ionized Calcium binding adaptor molecule 1
IFN-γ: Interferon-γ
IL-1β: Interleukin-1β
IL-6: Interleukin-6
iNOS: Inducible nitric oxide synthase
MAP2: Microtubule-associated protein 2
MerTK: Mer tyrosine kinase
NF-κB: Nuclear factor kappa-B
PD: Parkinson’s disease
PFF: Preformed fibrils
Rac1: Rac family small GTPase 1
RhoA: Ras homolog family member A
scFv: Single-chain variable fragment
TGF-β: Transforming growth factor-β
TH: Tyrosine hydroxylase
TNF-α: Tumor necrosis factor-α
