# Supplementary material for: Attenuating α-synuclein pathology in mice with in situ engineered astrocytes
Source: Transl Neurodegener. 2025 Nov 20;14:58. doi: 10.1186/s40035-025-00518-0 (PMC12632020; doi:10.1186/s40035-025-00518-0)
Supplement: Supplementary file 1 — Additional file 1. Fig. S1 The expression of CAR in vitro. Fig. S2 The effect of CAR-A on the neurons and microglia in vitro. Fig. S3 CAR expression did not interfere the physiological function of astrocytes. Fig. S4 Establishment of PFF-seeded A53T mouse model and the expression of CAR in vivo. Fig. S5 CAR decreased pathology in PFF-seeded A53T mice. [file 40035_2025_518_MOESM1_ESM.docx]

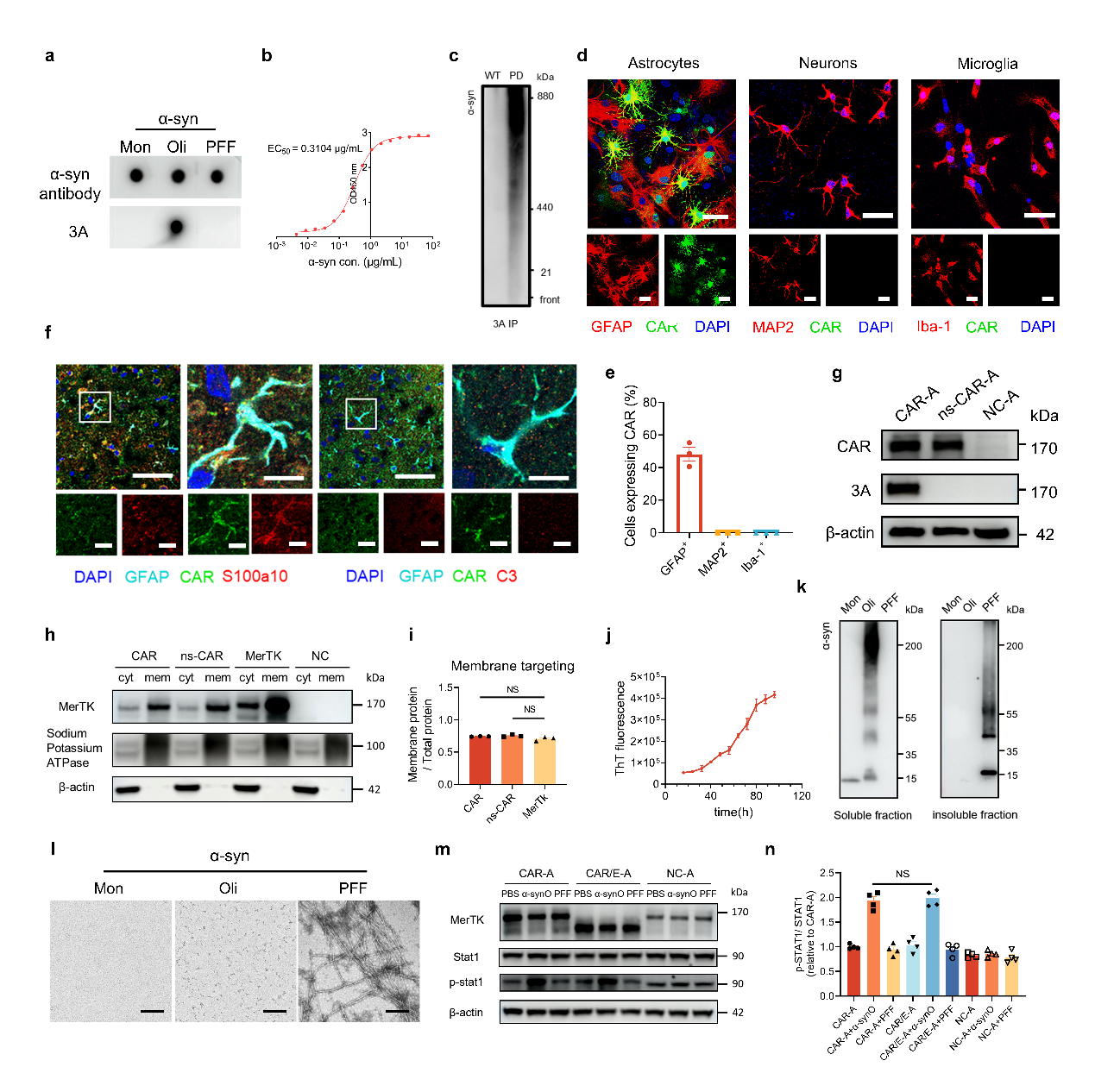


**Fig. S1 The expression of CAR *in vitro*. a,** The binding of 3A with different types of α-syn. Monomer, oligomer and PFF of α-syn were applied to nitrocellulose membrane, and then was probed by 3A and a control antibody against α-syn. Monoclonal antibody against α-syn was used as control. **b,** The affinity of 3A with α-synO. **c**, α-synO in brain lysates of PD transgenic mice. Brain lysates of PD transgenic mice were obtained by homogenizing brains in the lysis buffer (10 mM Tris-HCl, pH 7.4, 150 mM NaCl, 5 mM EDTA, 1% triton, protease inhibitor cocktails and phosphatase inhibitor cocktails) and centrifugation. The obtained supernatant was used for immunoprecipitation. α-synO in brain lysates of PD transgenic mice was immunoprecipitated with 3A, and then detected via western-blot using native-PAGE and an anti-α-synuclein monomer antibody. **d,** Representative images depicting the expression of CAR in astrocytes (left), neurons (middle) and microglia (right). Scale bars: 40 μm. **e,** Statistical analysis of the proportion of different cell types expressing CAR using Image J. *n* = 3 independent experiments. **f,** Representative images of CAR expression in A1-type astrocytes (C3 staining) and A2-type astrocytes (S100a10 staining) in mouse brains. Scale bars, 60 μm (low-magnification images) and 10 μm (high-magnification images), respectively. **g,** The intact expression of CAR on astrocytes. Membrane proteins extracted from ns-CAR-A and CAR-A were analyzed by western blotting using anti-3A and anti-MerTK antibodies. **h,** MerTK levels in the same volume of membrane (mem) and cytoplasmic (cyt) protein solution analyzed by western blotting. Astrocytes were transfected with plasmids expressing CAR, ns-CAR, wild type MerTK or empty plasmid. Sodium Potassium ATPase was used as the control of membrane proteins, and β-actin was used as the control of cytoplasmic proteins. **i,** Analysis the proportion of receptors expressed on the cell membrane in total MerTK protein by Image J. *n* = 3 independent experiments. **j,** α-syn aggregation detected by ThT fluorescence assay. **k,** The formation of α-synO and PFF verified by western blotting assay. α-syn monomers were solubilized in PBS at the concentration of 5mg/mL and incubated at 37 °C shaking for 7 days. α-syn in different aggregated forms in Triton X-100 soluble and insoluble fraction were detected by western blotting using an anti-α-syn monomer antibody. **l,** Representative TEM images of α-synMon, α-synO, α-synPFF. Scale bar: 200 nm. **m,** p-stat1 levels in astrocytes expressing CAR with or without EGFP fusion in the presence or absence of α-synO. Stat1 (in p-stat1) and β-actin were used as controls. **n,** Quantification of p-stat1 (**m**) using Image J. *n* = 4 independent experiments. Data are mean ± S.E.M. One-way ANOVA followed by Tukey’s multiple comparison tests was conducted for statistical analyses. NS indicates not significant.


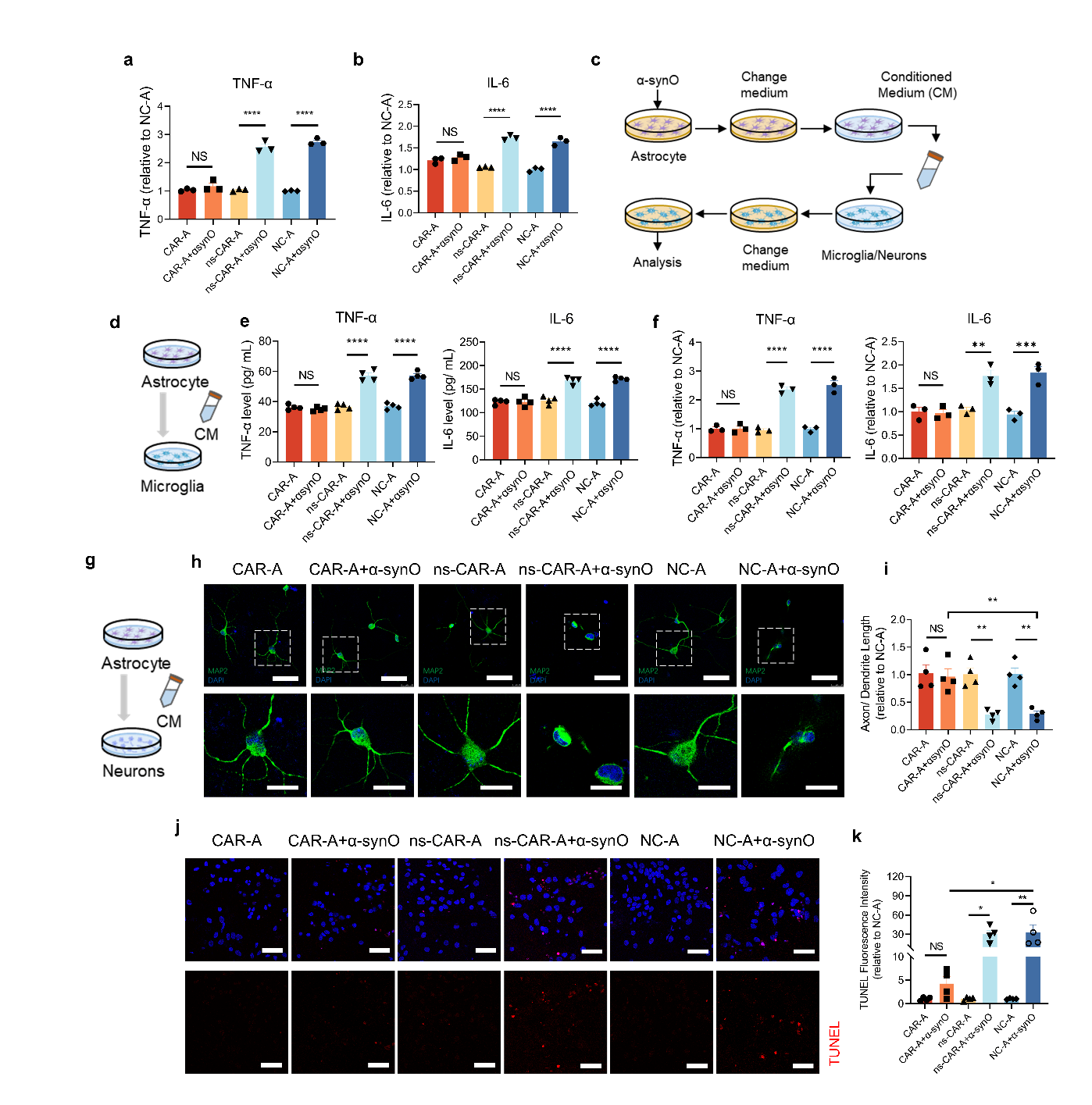


**Fig. S2 The effect of CAR-A on the neurons and microglia *in vitro*.** **a-b,** The mRNA levels of pro-inflammatory cytokines TNF-α (**a**) and IL-6 (**b**) in different groups of astrocytes determined by RT-PCR. *n* = 3 independent experiments. **c,** Cartoon depicting the experimental paradigm for identifying the microenvironmental influences in microglia and neurons by CAR-A using conditional medium (CM). **d-f,** CAR-A, ns-CAR-A or NC-A were treated with or without α-synO for 24 h. After replacing the medium and culturing for another 24 h, the CM was collected and added to microglia for 24 h. Then the medium was replaced and microglia were cultured for another 24 h (**d**). The pro-inflammatory cytokines (TNF-α and IL-6) secreted by microglia were determined by ELISA (**e**) and RT-PCR (**f**). *n* = 4 independent experiments (**e**) and *n* = 3 independent experiments (**f**). **g-i**, CAR-A, ns-CAR-A or NC-A were treated with or without α-synO for 24 h. After replacing the medium and culturing for another 24 h, the CM was collected and added to the medium of neurons for 24 h culture (**g**), and the morphology of the neurons was imaged by confocal microscopy (**h**). Scale bar, 50 μm (low-magnification images) and 20 μm (high-magnification images), respectively. The length of axons and dendrites were quantified using Image J (**i**). *n* = 4 independent experiments. **j-k,** The levels of apoptosis in CM-treated neurons measured by TUNEL assay. Apoptotic signals of the neurons were imaged by confocal microscopy (**j**). Scale bar, 50 μm. TUNEL fluorescence intensity was quantified using Image J (**k**). *n* = 4 independent experiments. Data are mean ± S.E.M. One-way ANOVA followed by Tukey’s multiple comparison tests was conducted for statistical analyses. **P* < 0.05, ***P* < 0.01, *****P* < 0.0001 indicate significance compared to respective groups. NS indicates not significant.


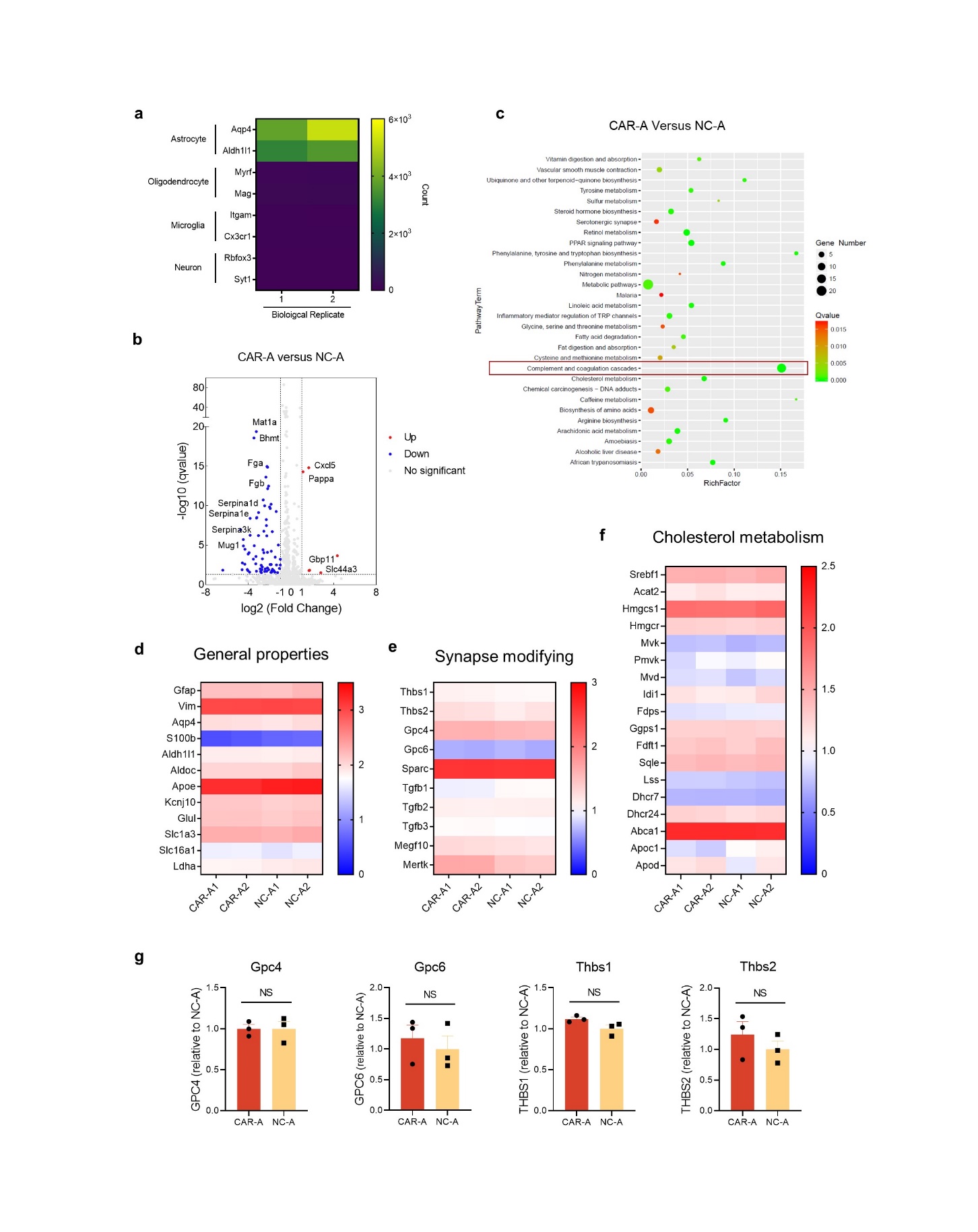


**Fig. S3** **CAR expression did not interfere the physiological function of astrocytes**. **a,** Gene expression level of different cell markers in the sample detected by RNA-seq (shown in count). RNA-seq data showed high expression level of astrocyte markers and low expression level of markers for other CNS cell types **b,** Volcano plot of differentially expressed genes between CAR-A and NC-A (p value ≤ 0.05 and fold change > 2). **c**, KEGG analysis for CAR-A and NC-A. **d-f,** Gene expression levels were normalized to log10 (FPKM+1) and shown in heat maps. The genes patterns involved in general properties of astrocytes (**d**), cholesterol metabolism (**e**), and regulation of neuronal synapse formation, function, and elimination (**f**) in CAR-A and NC-A were analyzed. **g,** The mRNA levels of Gpc4, Gpc6, Thbs1 and Thbs2 in different groups of astrocytes determined by RT-PCR. Data are mean ± S.E.M. *n* = 3 independent experiments. An unpaired t test with two-tailed was used for statistical analysis. NS indicates not significant.


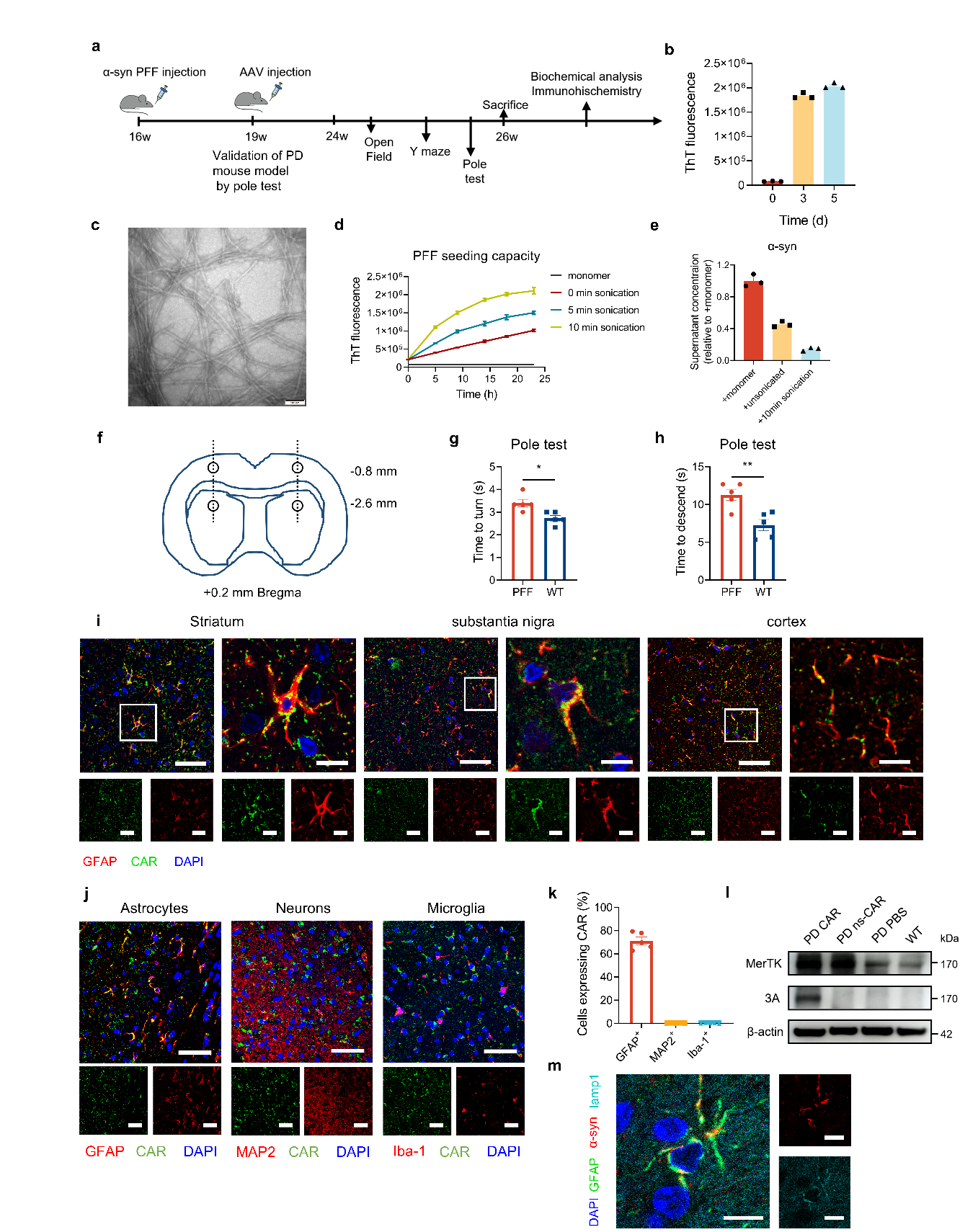


**Fig. S4 Establishment of PFF-seeded A53T mouse model and the expression of CAR *in vivo*. a**, Schematic diagram of stereotaxic injection of α-syn PFF and AAV, behavioral tests and the physiological and biochemical analysis for A53T mice. **b,** α-syn aggregation detected using ThT fluorescence assay. *n* = 3 independent experiments. **c,** Representative TEM images of α-syn PFF samples. Scale bar: 100 nm. **d-e,** α-syn monomer aggregation induced by PFF. PFF with or without sonication were added to α-syn monomer, ThT fluorescence assay (**d**) and the determination of supernatant concentration (**e**) were used to monitor the aggregation status of monomers. *n* = 3 independent experiments. **f,** Schematic representation of the α-syn PFF injection site. 5 μL (1.25 μL per injection site) of α-syn PFF was deposited into the somatosensory cortex region and dorsal neostriatum region in both the hemispheres of the brain. **g-h,** Motor ability of mouse model. The motor function of PFF-seeded A53T mice was tested via pole test 3 weeks after inoculation. The time for the mouse to turn on the pole and face downwards (**g**) and to descend from top to bottom of the pole (**h**) were recorded. *n* = 5 biologically independent animals. **i,** Representative images of astrocyte-specific expression of CAR in mouse striatum, substantia nigra and cortex. Scale bars, 40 μm (low-magnification images) and 10 μm (high-magnification images), respectively. **j,** Representative images depicting the specific expression of CAR in astrocytes rather than neurons and microglia in striatum of mouse brain. Scale bars: 40 μm. **k,** Statistical analysis of the proportion of different cell types expressing CAR using Image J. *n* = 5 biologically independent animals. **l,** The expression of CAR *in vivo*. Proteins extracted from striatum of mice were analyzed by western blotting using anti-3A and anti-MerTK antibodies, respectively. **m,** Representative images of α-syn collocated with lysosomes. Scale bars: 10 μm. Data are mean ± S.E.M. Unpaired two-tailed Student’s *t*-tests were used for statistical analyses (**g-h**). **P* < 0.05, ***P* < 0.01 indicate significance compared to respective groups.

**
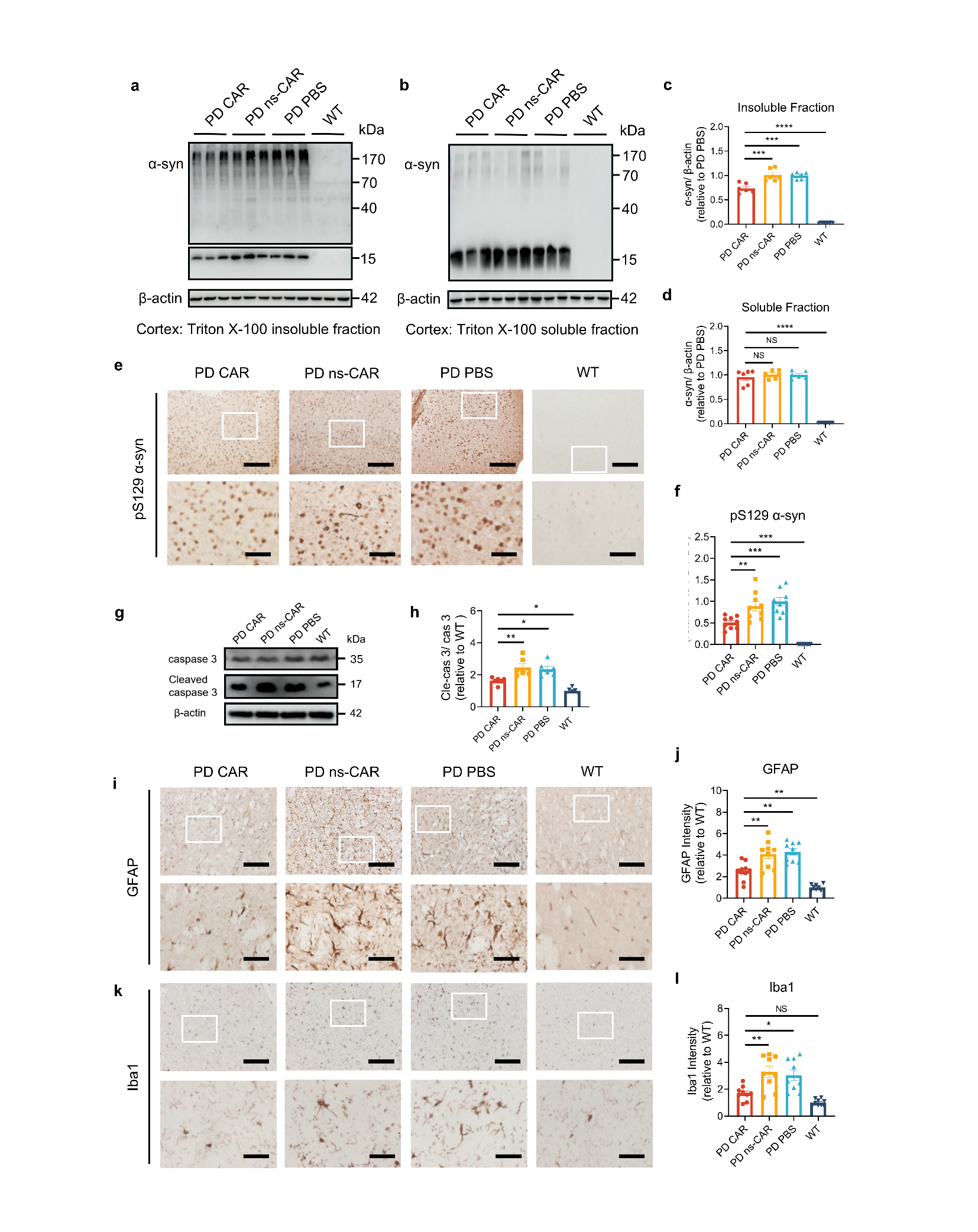
**

**Fig. S5 CAR decreased pathology in PFF-seeded A53T mice. a-b,** Total α-syn levels in Triton X-100 insoluble (**a**) and soluble (**b**) fractions of cortex in PFF-seeded A53T mice treated with or without CAR assessed by western blotting. β-actin was used as a control. **c-d,** Quantification of α-syn levels in (**a**) and (**b**) using Image J. *n* = 9 biologically independent animals. **e-f,** The levels of pS129 α-syn in cortex assessed by immunostaining (**e**), and quantified by Image J (**f**). Scale bar, 200 μm (low-magnification images) and 50 μm (high-magnification images), respectively. *n* = 4 biologically independent animals. **g,** Cleaved caspase 3 levels in the brainstem measured by western blotting. β-actin and caspase 3 were used as controls. **h,** Quantification of cleaved caspase 3 in (**g**) using Image J. *n* = 6 biologically independent animals. **i,** Representative images of astrocyte in striatum of different group mice detected by anti-GFAP antibody. **j,** The levels of GFAP in (**i**) were quantified by Image J. Scale bar, 200 μm (low-magnification images) and 50 μm (high-magnification images), respectively. *n* = 9 biologically independent animals. **k,** Representative images of microglia in striatum of different group mice detected by anti-Iba-1 antibody. **l,** The levels of Iba-1 in (**k**) quantified by Image J. Scale bar, 200 μm (low-magnification images) and 50 μm (high-magnification images), respectively. *n* = 9 biologically independent animals. Data are mean ± S.E.M. One-way ANOVA followed by Tukey’s multiple comparison tests was conducted for statistical analyses. **P* < 0.05, ***P* < 0.01, ****P* < 0.001, *****P* < 0.0001 indicate significance compared to respective groups. NS indicates not significant.
